# Erythropoiesis–inosine metabolic axis failure underlying retinal neurodegeneration in glaucoma: novel diagnoses and therapies

**DOI:** 10.1038/s12276-026-01654-x

**Published:** 2026-02-13

**Authors:** Yuyu Chou, Wuping Liu, Yanxiu Li, Changhan Chen, Cheng Luo, Shiping Shen, Piaoyu Dai, Lemeng Feng, Wenhao Xiao, Yiyan Wang, Juncheng Wang, Linlin Wan, Zhiyu Yang, Tingting Xie, Yujin Zhang, Rodney E. Kellems, Weitao Song, Xiaobo Xia, Yang Xia

**Affiliations:** 1 Eye Center of Xiangya Hospital, Central South University, Changsha, China; 2 National Medical Metabolomics International Collaborative Research Center, Xiangya Hospital, Central South University, Changsha, China; 3 Hunan Key Laboratory of Ophthalmology, Changsha, China; 4 National Clinical Research Center for Geriatric Disorders, Xiangya Hospital, Central South University, Changsha, China; 5 Department of Otolaryngology Head and Neck Surgery, Xiangya Hospital, Central South University, Changsha, China; 6 Otolaryngology Major Disease Research Key Laboratory of Hunan Province, Xiangya Hospital, Central South University, Changsha, China; 7 Department of Radiology, Xiangya Hospital, Central South University, Changsha, China; 8 Department of Biochemistry and Molecular Biology, The University of Texas McGovern Medical School, Houston, TX USA

**Keywords:** Neurodegenerative diseases, Retina, Haematopoietic stem cells

## Abstract

Glaucoma, long considered an ocular-limited, age-dependent and hypoxia-driven neurodegeneration, is here reframed as a systemic erythroid–inosine axis failure that originates in the bone marrow yet culminates in retinal ganglion cell (RGC) death. By mining UK Biobank datasets (*n* = 127,028) and validating our findings in an independent clinical cohort (*n* = 178), we reveal that glaucoma is preceded by dyserythropoiesis and a compensatory, AMPK-driven metabolic rewiring of mature erythrocytes that hypercatabolizes inosine to enhance oxygen unloading. This adaptation collapses when accelerated erythrocyte inosine metabolism drains systemic pools, starving high-energy demand hematopoietic progenitors, driving retinal microenvironment hypoxia and accelerating RGC loss. Genetic ablation of murine erythroid equilibrative nucleoside transporter 1 (ENT1) recapitulates the hallmark features of patients with glaucoma, including impaired erythropoiesis, reduced oxygen delivery, retinal hypoxia and RGC apoptosis in both age and intraocular pressure-induced glaucoma models. Conversely, inosine repletion reconstitutes erythroid output, restores oxygen delivery from mature erythrocytes and halts neurodegeneration in inducible glaucoma models. A ten-metabolite erythrocyte signature centered on inosine metabolism offers diagnostic potential. Altogether, our work redefines glaucoma as the first treatable systemic erythroid-driven hypoxic syndrome, positioning inosine as a pleiotropic metabolic rescue factor for neurodegeneration and a powerful biomarker for intercepting hypoxia-driven pathologies across organs.

## Targeting inosine offers new hope for glaucoma treatment

Glaucoma is a major cause of blindness. It involves damage to the optic nerve and is often linked to high eye pressure. However, even with controlled pressure, vision can worsen. Recent studies suggest that systemic issues, such as problems with red blood cells (RBCs), might play a role. Researchers studied RBCs in patients with glaucoma to understand their role in the disease. They analyzed blood samples from patients and healthy individuals to compare RBC function and metabolism. They found that patients with glaucoma had fewer RBCs and these cells showed signs of metabolic stress. The study revealed that RBCs in patients with glaucoma have altered metabolism, relying more on a compound called inosine for energy. This change might be a response to low oxygen levels in the eye. The findings suggest that improving RBC function could help manage glaucoma.

This summary was initially drafted using artificial intelligence, then revised and fact-checked by the author.

## Introduction

Glaucoma is the leading cause of irreversible blindness worldwide, affecting over 95 million people globally^1^. With the global population aging, the prevalence of glaucoma is projected to rise to 118 million by 2040^2^, underscoring an urgent need for more effective treatment strategies. As a progressive optic neuropathy characterized by the degeneration of retinal ganglion cells (RGCs)^3^, glaucoma has long been managed primarily through lowering intraocular pressure (IOP). However, even with well-controlled IOP, many patients still experience visual deterioration^4^, suggesting that pressure-independent mechanisms contribute to disease progression.

Emerging epidemiological studies have linked glaucoma to systemic comorbidities such as diabetes, hypertension and cardiovascular disease^5–7^, implying that glaucoma may reflect not only localized ocular pathology but also systemic physiological dysregulation. RGCs are metabolically demanding neurons that rely heavily on continuous oxygen and glucose supply for energy homeostasis, and notably, the retina exhibits one of the highest oxygen consumption rates per tissue weight in the human body^8^. Consistent with this vulnerability, previous studies showed that elevated IOP leads to increased expression of hypoxia-inducible factors (HIFs) in the retina, the optic nerve and even the superior colliculus^9,10^. Hypoxia-induced mitochondrial dysfunction, excessive activation of inflammatory responses, and pathological alterations in signaling pathways—including the CASP8–HIF-1α–NLR and Wnt/β-catenin pathways—can all contribute to RGC death^11–14^ and exacerbate disease progression. More recently, mitochondrial respiratory capacity and systemic levels of nicotinamide adenine dinucleotide (NAD) were found to be markedly reduced in patients with glaucoma, correlating closely with visual field deterioration^15^. Notably, early transcriptomic and metabolomic analyses in preclinical glaucoma models revealed that retinal glucose accumulation and pyruvate decrease before observable RGC loss, implicating an early breakdown in glycolytic flux^16^. These findings underscore the importance of systemic and retinal metabolic disorders as one of the driving forces in glaucomatous neurodegeneration. Overall, a growing body of evidence indicates that hypoxia, redox imbalance and energy failure are extremely important for glaucoma pathogenesis, yet the specific cell types and underlying mechanisms causing systemic or optical hypoxia with disturbed metabolism and progression of glaucoma remain unclear.

One overlooked systemic contributor may be erythrocytes, which account for ~83% of all human cells and serve as the sole oxygen carriers and major nutrition consumers in the circulation, such as glucose^17^. In addition to delivering oxygen, erythrocytes dynamically shape inter-organ metabolic communication by regulating tissue oxygenation and nutrient availability. The ability of erythrocytes to meet tissue metabolic demands and to counteract tissue hypoxia is governed by both their production (erythropoiesis) and metabolic adaptability. Given their central role in oxygen–glucose balance, particularly under hypoxic stress, erythrocytes may represent an unrecognized axis linking systemic dysfunction to glaucomatous neurodegeneration. Notably, in other hypoxia-related conditions, such as high-altitude adaptation^18^ and chronic kidney disease^19^, mature erythrocytes exhibit glucose metabolic reprogramming to enhance oxygen release. In parallel, stress erythropoiesis can be triggered by metabolites such as glucose and glutamine^20,21^. Whether these dual compensatory responses—erythrocyte metabolic reprogramming and enhanced production—also occur in glaucoma remains unknown. Moreover, no studies have systematically investigated how erythropoiesis, mature erythrocyte metabolism and RGC vulnerability interact in the context of glaucoma.

To address this gap, we conducted a comprehensive investigation integrating data from two independent cohorts. We analyzed red blood cell (RBC) number, function and metabolic features in patients with glaucoma, and explored small-molecule metabolic signatures as potential diagnostic biomarkers and therapeutic targets. Our findings reported here reframe glaucoma as a systemic, circulation-linked metabolic disorder driven in part by erythropoietic failure and erythrocyte maladaptation, and provide new insights into the systemic mechanisms contributing to retinal neurodegeneration.

## Materials and methods

### Human participants

The current study consecutively enrolled patients with glaucoma and age- and sex-matched healthy individuals at the Department of Ophthalmology of Xiangya Hospital, Central South University at Changsha, Hunan, China, from July 2022 and October 2022. Written informed consent was obtained from all participants. The clinical diagnosis of glaucoma was confirmed by two experienced glaucoma specialists, each with over 20 years of clinical practice, ensuring diagnostic accuracy and consistency. Peripheral blood samples (5 ml) were collected. In addition, we comprehensively reviewed patients with glaucoma and age- and sex-matched healthy controls over the past year. The medical records of all participants were extracted, including demographic information, chief complaint, self-reported disease duration from symptom onset to diagnosis of glaucoma, visual acuity (VA), IOP, cup-to-disc ratio (C/D) ratio, visual field test, peripapillary retinal nerve fiber layer (pRNFL) thickness and complete blood count (CBC) test results related to erythrocytes.

According to the World Health Organization classification, patients who met the primary open-angle glaucoma (POAG) or primary angle-closure glaucoma (PACG) diagnostic criteria were enrolled in this study (ICD-10 code H40.1 and H40.2). Exclusion criteria for all participants were as follows: (1) participants with systemic diseases affecting the number and function of RBCs, such as anemia, hemophilia, leukemia and chronic kidney disease; (2) participants with diseases affecting whole-body metabolisms, such as diabetes, tumors and hyperthyroidism; (3) participants with other ophthalmic comorbidity affecting retinal microcirculation or structure such as age-related macular degeneration, retinal vein occlusion and diabetic retinopathy; (4) participants with secondary glaucoma due to other causes; and (5) pregnant women and lactating women. In accordance with the Declaration of Helsinki tenets, this study was approved by the Central South University Committee for the Protection of Human Subjects (2025020283).

### Animals

All animal procedures were conducted following the guidelines for the use of experimental animals and were approved by the Animal Care and Use Committee at Central South University (license number CSU-2024-0272). The experiments were performed on 8–10-week-old C57BL/6J male mice and erythrocyte-specific deletion of equilibrative nucleoside transporter 1 (ENT1) mice^22^, weighing approximately 20 g. Erythrocyte-specific deletion of *Ent1* was generated by crossing mice homozygous for a floxed *Ent1* allele with EpoR-Cre mice. The C57BL/6J mice were obtained from Hunan SJA Laboratory Animal Co., Ltd (Changsha, Hunan, China) (license number SYXK (Xiang) 2020-0019). The animals were maintained under controlled conditions, with a 12-h cycle of light and dark at a temperature of 22 °C, and had access to food and water ad libitum.

### Metabolomic profiling

Erythrocytes and plasma were lysed with lysis solution (methanol:acetonitrile:water 5:3:2). The mixtures were then vortexed continuously for 30 min at 4 °C, followed by centrifugation at 18,213*g* for 10 min at 4 °C. Equal volumes of supernatants were injected into the ultra-high-performance liquid chromatography–mass spectrometry system using a Vanquish UHPLC coupled to a Q Exactive mass spectrometer (Thermo Fisher). The 5-min gradient was utilized to analyze samples as previously described^23,24^. Raw data files were converted to mzXML format by RawConverter (Scripps Research Institute). The metabolomic data were analyzed via Maven (Princeton University, Princeton, NJ) and normalized on the basis of the website MetaboAnalyst (https://www.metaboanalyst.ca/MetaboAnalyst/). Metabolomics data were normalized to RBC extraction volumes and mean corpuscular volume (MCV).

### Isotopic-labeled flux

Human blood collected in heparin tubes was centrifuged at 1,000*g* for 10 min at room temperature. Erythrocytes were purified and washed three times with F-10 Nutrient Mix (without glucose, Procell), and resuspended to a hematocrit (HCT) of 4%. Erythrocytes (1 ml) were added to each well of a 12-well plate and incubated with 1 mM of either ^13^C_6_-labeled glucose (Sigma-Aldrich) or ^13^C_5_-ribose-labeled inosine (MedChemExpress) for 30 min, 2 h or 6 h with continuous shaking. After incubation, erythrocytes and supernatants were collected, lysed as previously described^19,22^ and analyzed using a Vanquish UHPLC system coupled to a Q Exactive MS (Thermo Fisher). Metabolite identification and isotopologue distribution analysis were performed with Maven.

### Western blot

Western blot analysis of erythrocytes was performed as described previously^25^. Frozen erythrocytes were lysed in cold water on ice for 10 min at a 1:10 ratio, in the presence of 1× protease inhibitor cocktail (Selleck, B14001) and 1× phosphatase inhibitor cocktail (Selleck, B15001). The lysate was then centrifuged at 2,000*g* for 10 min at 4 °C. After discarding the supernatant, the pellet was resuspended in cold 1× phosphate-buffered saline (PBS). This centrifugation step was repeated approximately six times until the pellet color changed from red to white. The final pellet was resuspended in RIPA buffer, vortexed for 30 min at 4 °C and sonicated for 10 s. The lysate was then centrifuged again, and the supernatant was collected. These supernatants contained the erythrocyte membrane proteins, which were quantified using the BCA Protein Assay Kit (Thermo Scientific). Samples were boiled in Laemmli sample buffer and stored at −80 °C for future use. For western blot analysis, the samples were loaded onto a 10% SDS–PAGE gel, and the membranes were incubated with primary antibodies against ENT1 (Proteintech, 11337-1-AP, 1:1,000), GLUT1 (Abcam, ab115730, 1:5,000) and β-actin (Proteintech, 66009-1-Ig, 1:3,000), followed by incubation with appropriate secondary antibodies.

### Purine nucleoside phosphorylase (PNP) activity measurement

The erythrocytes were 1:1 added into PBS with 0.3% Triton X-100. The activity of PNP was assessed spectrophotometrically by adding 1 μl of the cell lysate to 200 μl of the reaction buffer, which consisted of 50 mM potassium dihydrogen phosphate (pH 7.5), 1 mM inosine and 10 mU of xanthine oxidase (Sigma). The reaction progress was monitored at 293 nm for 30 min at 25 °C. Subsequently, the sample hemoglobin (Hb) concentration was quantified by measuring absorption at 414 nm, and the PNP activity was normalized to the Hb concentration.

### Measurement of P50

After the Bloodox-2019 portable oxygenation function detection system is powered on and the membrane is activated, 4 ml of P50 buffer and 20 µl of EDTA-treated fresh whole blood are added to the sample pool. Once the temperature reaches 37 °C and the oxygen supply is stabilized, measurements commence. The oxygen dissociation curve is then obtained, and the oxygen partial pressure at 50% Hb saturation (P50) is determined.

### Retinal ischemia–reperfusion (I/R) mouse model

The mouse model of pathologically high IOP was established by increasing the anterior chamber pressure with a saline-perfusion system as previously described^26,27^. After anesthetization, pupil dilation and corneal anesthetization, mice were placed under the surgical microscope. A 30-gauge cannula needle was gently inserted into the anterior chamber, and the pressure was gradually increased to 120 mm Hg by adjusting the saline bottle height (162 cm) and maintaining it for 90 min. For the *eEnt1*^*−/−*^ and *Ent1*^*f/f*^ mice, we reduced the molding duration and the anterior chamber pressure to distinguish the differences between the six groups better by adjusting the saline bottle height (150 cm) and maintaining it for 45 min.

### Erythrocyte lifespan measurement

As previously described^28^, the lifespan of erythrocytes was measured using flow cytometry. The EZ-Link NHS-Biotin (Thermo Fisher Scientific, catalog number 20217) was administered via the retro-orbital plexus. Blood samples from mice were collected before I/R modeling (day 0) and at various time points after I/R modeling (3, 7, 14, 21 and 28 days after NHS-Biotin injection), and stained with streptavidin and Ter-119. The proportion of streptavidin-positive to Ter119-positive cells was measured.

### Flow cytometry analysis

Flow cytometry analysis was performed as described previously^29^. For the preparation of a single-cell suspension from the spleen, approximately one-third of the spleen was dissected. A clean culture dish was placed with a filter, and 1 ml of flow buffer (1% bovine serum albumin and 1 mM EDTA in 1× PBS) was used to wet the filter. The spleen tissue was then placed in the filter, and gentle grinding was performed in a concentric circular motion. The grinding was continued until only connective tissue remained, after which 3 ml of flow buffer was added to wash the filter. The total liquid was centrifuged and resuspended in 1× PBS. For the preparation of a BM single-cell suspension, cells were isolated from the tibia and femur of mice and suspended in 1× PBS. The single-cell samples were incubated with the viability marker Zombie Aqua (BioLegend 423101, 1:500) for 15 min at room temperature in the dark. Afterward, the cells were washed with flow buffer and stained with either Mix I or Mix II for 30 min at 37 °C in the dark. All antibodies were used at a concentration of 1:100. Following staining, the cells were washed with flow buffer and resuspended in 100 µl of flow buffer, and analyzed within 1 h using spectral flow cytometry (Cytek Northern Lights). Mix I included Pacific Blue-conjugated lineage markers (BioLegend 133310), c-KIT (BD 553355), Sca-1 (BioLegend 108134), CD16/32 (BD 570676), CD34 (BD 560230), CD135 (BD 562537) and CD127 (BD 560733). Mix II included CD45 (BD 561037), CD11b (Thermofisher 56-0112-80), Gr-1 (BioLegend 108433), Ter119 (BioLegend 116208) and CD44 (BioLegend 103041). The data were analyzed by FlowJo software (V10.8.1).

### HSPC culture to induce erythroid cells and flow cytometry

Bone marrow cells were collected from the femurs and tibias of wild-type (WT) mice, as well as *Ent1*^*f/f*^ and *eEnt1*^*−/−*^ mice, aged 8–10 weeks. Single-cell suspensions were obtained by passing the cells through a cell strainer. Subsequently, hematopoietic stem/progenitor cells (HSPCs) were enriched through negative selection utilizing a mouse hematopoietic progenitor cell enrichment kit (BD 558451). In brief, the isolated bone marrow cells were incubated with a series of biotin-conjugated lineage antibodies, including CD3, CD11b, CD45R, Ly-6G/C and TER119, followed by incubation with streptavidin magnetic beads. The differentiated cells expressing these lineage markers were separated from the undifferentiated HSPCs using a magnetic bar. The enriched HSPCs, which lacked these lineage markers, were further isolated by centrifugation. Purified HSPCs were then seeded in wells at a density of 1.5 × 10^5^ cells ml^−1^ and cultured as previously described, with modifications for erythroid cell differentiation^30^. Cultured murine HSPCs from WT, *Ent1*^*f/f*^ and *eEnt1*^*−/−*^ mice were treated with or without inosine and forodesine (foro, a PNP inhibitor). The percentage of erythroid cells was determined by flow cytometry using CD71 and Ter119 antibodies.

### Statistical analyses

Continuous variables were reported as the mean ± standard deviation (s.d.). Normality was assessed using visual inspection (histograms and probability plots), the D’Agostino Pearson omnibus test and the Kolmogorov–Smirnov test. Depending on the normality findings, two-tailed unpaired *t*-tests or Mann–Whitney *U* tests were used to compare differences among various groups. Pearson or Spearman correlation analysis was performed according to the data normality. *P* values less than 0.05 were considered statistically significant. Statistical analyses were performed using SPSS software (SPSS for Mac, version 23.0; IBM/SPSS), and GraphPad Prism 10 (GraphPad Software). The schematic diagram was created with BioRender.com.

## Results

### Abnormalities in the number, function and metabolism of erythrocytes of patients with glaucoma in two independent large human cohorts

**Fig. 1:**
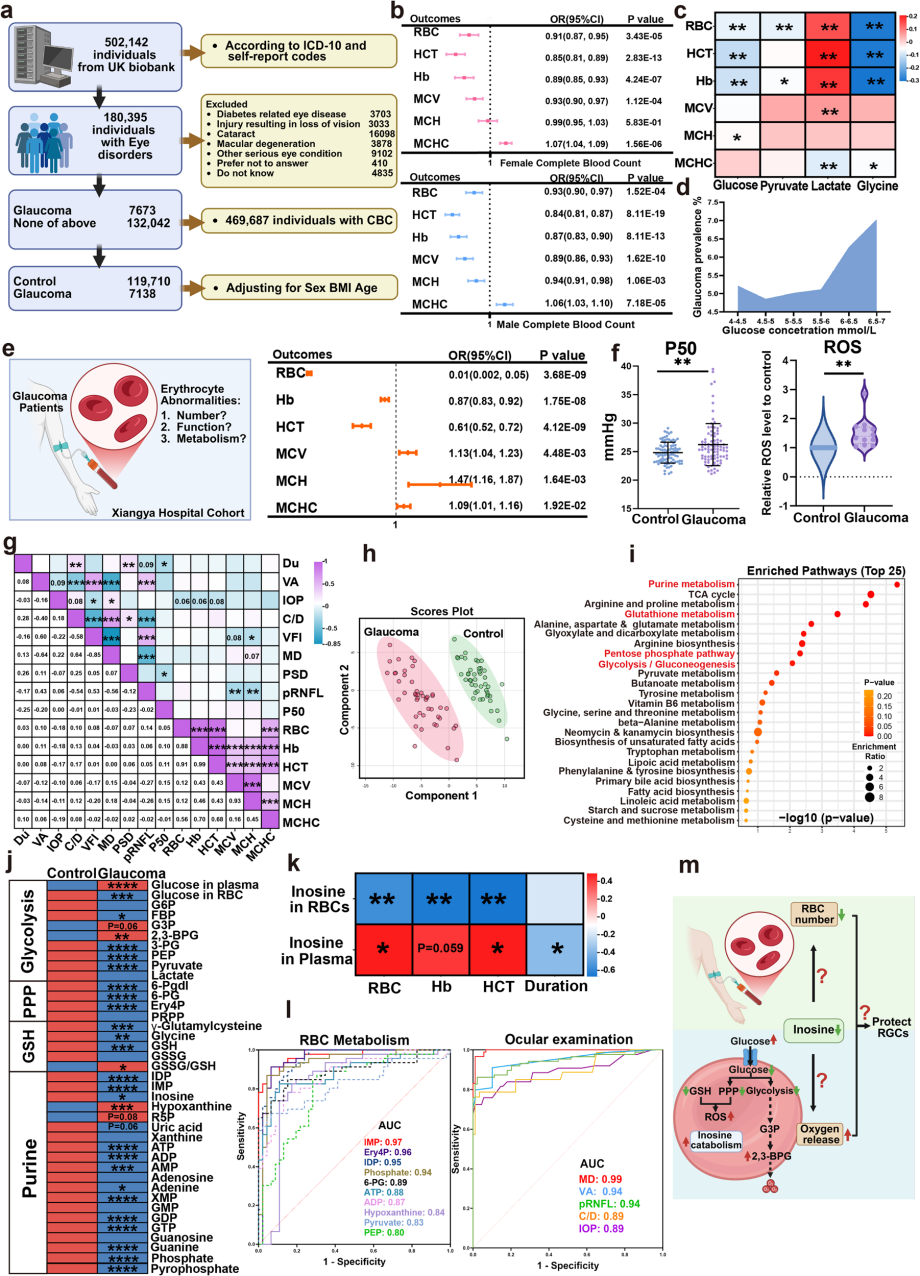
Abnormalities in the number, function and metabolism of erythrocytes of patients with glaucoma. **a**, Inclusion and exclusion criteria of the participants in the UK Biobank. **b**, Relationship between erythrocytes and the prevalence of glaucoma from the UK Biobank. **c**, Correlation analysis between erythrocyte parameters and the level of glucose, pyruvate, lactate and glycine in plasma from the UK Biobank. **d**, Relationship between glaucoma prevalence and the blood glucose concentration from the UK Biobank. **e**, Relationship between erythrocytes and the prevalence of glaucoma in the Xiangya Hospital cohort. **f**, Comparison of P50 values and the level of ROS in erythrocytes between healthy controls and patients with glaucoma. **g**, Correlation analysis between erythrocyte parameters and visual function and ocular structure. **h**, PLS-DA of erythrocyte metabolism between patients with glaucoma and healthy controls. **i**, Pathway enrichment analysis. **j**, Heatmap of the erythrocyte intermediates of glycolysis, the PPP, the glutathione synthesis pathway and purine metabolism. Red represents an upregulated level, and blue represents a downregulated level. **k**, Relationship between the relative concentration of inosine within RBCs or in plasma and erythrocyte parameters, as well as disease duration. **l**, The top ten candidate biomarkers of glaucoma in erythrocyte metabolism and the established ocular diagnostic indicators. **m**, Scheme illustrating count reduction and metabolic disorder in the glaucoma mature erythrocyte. FBP, fructose 1,6-bisphosphate; PEP, phosphoenolpyruvate; 6-Pgdl, glucono-1,5-lactone 6-phosphate; 6-PG, 6-phospho-D-gluconate; Ery4P, erythrose 4-phosphate; PRPP, phosphoribosyl pyrophosphate; GSH, glutathione; GSSG, glutathione disulfide. Data are presented as mean ± s.d.; *P < 0.05, **P < 0.01, ***P < 0.001.

To precisely define the hematological changes in patients with glaucoma, we took advantage of the UK Biobank. In brief, a total of 180,395 individuals from the UK Biobank with eye problems were included in this project. After excluding participants with other ocular diseases, such as diabetes-related eye disease, injury resulting in loss of vision, macular degeneration and other serious eye conditions, and individuals without CBC test results, 7,138 individuals diagnosed with glaucoma and 119,710 individuals with none of the above diseases as controls were ultimately selected (Fig. 1a). To rigorously evaluate the independent contribution of each erythrocyte-related index to glaucoma risk, we performed multivariable logistic regression analyses stratified by sex and adjusted for age and body mass index (BMI). This analysis confirmed that RBC counts, Hb, HCT and MCV were independently protective against glaucoma, whereas mean corpuscular Hb concentration (MCHC) was associated with an increased risk of glaucoma (Fig. 1b).

While erythrocyte alterations have been implicated in glaucoma, it remains unclear whether these hematologic changes reflect or contribute to systemic metabolic disturbances. To explore this possibility, we compared plasma metabolomic profiles between age- and sex-matched glaucoma patients and healthy controls. Notably, within the glaucoma group, key erythrocyte parameters were significantly associated with specific circulating metabolites. RBC count, HCT and Hb negatively correlated with plasma glucose and glycine levels, and positively with lactate levels (Fig. 1c). Supporting this observation, analysis of the UK Biobank cohort revealed that, even after excluding individuals with diagnosed diabetes, hypoglycemia or extreme glucose levels (<4 mmol/l or >7 mmol/l), glaucoma prevalence increased with higher blood glucose concentrations (Fig. 1d). Altogether, the large-scale the UK Biobank dataset, which has excluded potential confounders such as age, sex and BMI, confirmed that the number of RBCs is decreased in patients with glaucoma and may be linked to metabolic dysregulation. However, due to the limited plasma metabolomic data in the UK Biobank, further investigation is warranted to delineate the metabolic mechanisms underlying these associations.

Given that RBCs act as sensors and carriers of oxygen, and in light of our intriguing findings showing strong correlations between reduced RBC count, HCT and Hb, as well as plasma glucose, glycine and lactate levels, with glaucoma prevalence in the UK Biobank, we were motivated to validate these findings, further assess the RBC functional changes, and define both RBC and plasma comprehensive metabolomic signatures with the goal of identifying new diagnostic biomarkers and potential therapies for glaucoma in our own well-characterized large human cohort. We constructed our own independent cohorts by enrolling 178 participants, including 89 patients (34 males and 55 females) with primary glaucoma and 89 sex–age-matched healthy controls. Similar to the findings from the UK Biobank, RBC numbers, Hb and HCT were significantly decreased in patients with glaucoma in our cohort, while MCV, mean corpuscular Hb (MCH) and MCHC increased (Table 1). The multivariable logistic regression analyses also demonstrated that RBC numbers, Hb and HCT were protective factors against glaucoma, while MCV, MCH and MCHC were related to an increased risk of glaucoma, after adjusting for hypertension, smoking status and cardiovascular risk, as well as sex and age (Fig. 1e). Thus, we demonstrated that decreased RBC number is a common pathogenic feature in two independent human glaucoma cohorts. Moreover, we specifically quantified RBC oxygen delivery capacity by measuring the oxygen tension corresponding to 50% Hb saturation (P50). Our findings indicated that the P50 level in glaucoma erythrocytes compensatively increased, and the level of reactive oxygen species (ROS) was also elevated (Fig. 1f). In addition, all of the patients with glaucoma were examined by ophthalmic tests, including VA, IOP, pRNFL thickness, C/D ratio and visual field, including pattern standard deviation (PSD), mean deviation absolute value (MD) and visual field index (VFI). Initial correlation analyses revealed that changes in ocular structure were congruent with the pattern of glaucoma disease, as indicated by lower VFI, larger MD and greater PSD associated with a bigger C/D ratio, higher IOP, reduced VA and longer disease duration. Furthermore, the thickness of pRNFL was positively correlated with VA and VFI, and negatively correlated with the C/D ratio and MD. Further correlation analyses of erythrocyte parameters with ocular function and structure revealed that lower P50 was linked to longer disease duration and larger PSD. Reductions in RBC count, Hb and HCT were associated with higher IOP, and larger MCV and MCH were associated with thinner pRNFL, lower VFI and bigger MD (Fig. 1g). Finally, to further explore subtype-specific patterns, we examined the distribution of glaucoma subtypes within our cohort: among the 89 patients with glaucoma, 64 were diagnosed with PACG and 25 with POAG. Despite anatomical and clinical differences between PACG and POAG, both subtypes consistently exhibited reductions in RBC count, Hb and HCT (Supplementary Fig. 1a) and an increase in P50 level (Supplementary Fig. 1b), indicating shared erythroid abnormalities between these two glaucoma subtypes. Our findings suggest that systemic erythroid defects may represent a common pathogenic feature underlying both major clinical forms of glaucoma. Thus, we provide evidence from two independent human glaucoma cohorts that reduced RBC number is a previously unrecognized pathogenic feature positively correlated with disease severity in patients with glaucoma, while RBC oxygen delivery capacity is induced and its induction is negatively correlated with disease severity. Our findings immediately raise a new but compelling possibility that increased RBC oxygen-releasing capability is a compensatory response to decreased RBC number and local ocular hypoxia to counteract RGC damage and progression of glaucoma.

**Table 1:** Demographic and clinical information of the Xiangya cohort.

Variable	Control (*N* = 89)	Glaucoma (*N* = 89)	*P* value
Age (years)	61.33 (15.08)	59.46 (13.42)	0.231
Sex (male/female)	55/34	55/34	
RBC (10^12^/l)	4.78 (0.34)	4.39 (0.49)	<0.001
Hb (g/l)	144.08 (12.06)	135.19 (12.43)	<0.001
HCT (%)	43.29 (3.42)	40.37 (3.51)	<0.001
MCV (fl)	90.49 (3.63)	92.42 (5.96)	0.010
MCH (pg)	30.11 (1.29)	30.89 (2.10)	0.003
MCHC (g/l)	332.74 (4.35)	334.21 (4.78)	0.034
WBC (10^9^/l)	6.23 (1.35)	6.26 (1.62)	0.894
N (10^9^/l)	3.62 (1.02)	3.97 (1.42)	0.065
L (10^9^/l)	1.96 (0.53)	1.68 (0.54)	0.001
NLR	1.96 (0.80)	2.64 (1.40)	<0.001
Plt (10^9^/l)	222.37 (54.46)	208.11 (56.16)	0.087
ALT (U/l)	21.42 (9.32)	21.58 (15.43)	0.936
AST (U/l)	24.10 (5.69)	25.06 (10.46)	0.453
TPA (g/l)	73.96 (3.22)	70.82 (5.36)	<0.001
ALB (g/l)	44.81 (2.42)	43.29 (3.59)	0.001
GLOB (g/l)	29.16 (2.92)	27.62 (3.46)	0.002
Urea (mmol/l)	4.94 (1.27)	5.61 (1.54)	0.002
UA (μmol/l)	357.12 (86.2)	336.65 (86.56)	0.121
Creatinine (μmol/l)	77.34 (14.72)	68.85 (25.29)	0.007
Hypertension (%)	11.24%	23.86%	0.027
Smoking (%)	19.10%	10.11%	0.089
Cardiovascular risk (%)	11.24%	12.36%	0.816
VFI (%)	Normal	30.23 (33.26)	
MD (dB)	Normal	24.29 (10.07)	
PSD (dB)	Normal	7.07 (3.22)	
C/D ratio	Normal	0.80 (0.23)	
pRNFL (μm)	Normal	58.85 (27.32)	
Duration (months)	–	24.85 (46.29)	

Data are expressed as mean (s.d.) or percentage. Depending on the normality findings, two-tailed unpaired *t*-tests or Mann–Whitney *U* tests were used to compare differences between groups. RBC, RBC number; WBC, white blood cell; N, neutrophils; L, lymphocyte; NLR, neutrophil to lymphocyte ratio; Plt, platelet; ALT, alanine aminotransferase; AST, aspartate aminotransferase; TPA, tissue plasminogen activator; ALB, serum albumin; GLOB, serum globulin; UA, uric acid; VFI, visual field index; MD, mean deviation absolute value; PSD, pattern standard deviation; C/D ratio, cup-to-disc ratio; pRNFL, peripapillary retinal nerve fiber layer thickness.

Next, we were prompted to define the comprehensive metabolomic signatures of erythrocytes and plasma in our own independent glaucoma human cohort by using untargeted high-throughput metabolomic profiling, including 92 participants (46 patients diagnosed with primary glaucoma and 46 sex- and age-matched healthy controls). The patient population included 24 with PACG and 22 with POAG, the two prevalent forms of glaucoma. Notably, metabolomic profiles from patients with PACG and those with POAG showed a similar alteration pattern in key metabolic pathways, including glycolysis, PPP and purine metabolism (Supplementary Fig. 1c). Given this high degree of overlap at both the hematologic and metabolic levels, we chose to integrate PACG and POAG data into a unified analysis to define a robust and coherent metabolic signature of glaucoma-associated RBC dysfunction that reflects the shared systemic pathology of the disease. A partial least-squares discriminant analysis (PLS-DA) revealed that erythrocyte metabolism substantially differed between glaucoma patients and healthy controls (Fig. 1h). The main differential metabolites were primarily enriched in the purine pathway, the pentose phosphate pathway (PPP), glycolysis and the glutathione synthesis pathway (Fig. 1i).

To gain deeper insight into the metabolic changes in RBCs associated with glaucoma, we conducted a comprehensive analysis of the principal metabolic pathways. The glycolysis pathway plays a pivotal role in erythrocytes, serving as the primary source of ATP and 2,3-bisphosphoglycerate (2,3-BPG), a negative allosteric regulator of Hb’s oxygen affinity, produced through a branch of glycolysis known as the Rapoport–Luebering shunt^31,32^. In comparison with healthy individuals, the levels of key metabolites in glycolysis are significantly reduced within erythrocytes of patients, potentially leading to a state of glucose starvation. This is further supported by the observation of increased glucose levels in the plasma, concurrent with a decrease in glucose inside erythrocytes (Fig. 1j). Regarding oxidative stress, both the PPP and the glutathione synthesis pathway within erythrocytes were reduced, and the ratio of oxidized to reduced glutathione increased in erythrocytes from glaucoma (Fig. 1j). These patients also exhibited significantly higher levels of ROS in their erythrocytes compared with healthy controls (Fig. 1f). Moreover, the purine pathway demonstrated a reduction in energy metabolism, manifesting significantly lower levels of ATP and GTP (Fig. 1j and Supplementary Table 1). Thus, we revealed that the erythrocytes of patients with glaucoma exhibit signs of glucose deprivation, increased oxidative stress and reduced energy metabolism. Our current cohort does not show major subtype-specific divergence. The shared metabolic reprogramming observed in both PACG and POAG underscores the systemic nature of erythroid dysfunction in glaucoma. However, future studies with larger, well-stratified cohorts will be valuable to determine whether subtle subtype-specific variations exist beyond this common metabolic core.

Notably, we observed a general downregulation of purine metabolism, except for an upregulation in inosine catabolism in glaucomatous RBCs. This resulted in decreased inosine levels and increased hypoxanthine and ribose-1-phosphate (R1P), the initial products of inosine catabolism. R1P is subsequently converted to ribose-5-phosphate (R5P), glyceraldehyde 3-phosphate (G3P) and 2,3-BPG. The levels of R1P/R5P and 2,3-BPG were elevated in glaucomatous RBCs, whereas other glycolytic metabolites were decreased (Fig. 1j). In addition, our results indicated that, among patients with glaucoma, RBC counts, Hb and HCT were negatively correlated with the level of inosine within RBCs, while they were positively associated with the level of inosine in plasma. Concurrently, erythrocyte and plasma inosine levels decreased with increasing disease duration (Fig. 1k). In view of the functional and metabolic changes of RBCs and their strong correlation with disease severity in patients with glaucoma, we were motivated to seek novel biomarkers of glaucoma from the erythrocyte perspective. The receiver operating characteristic curves were plotted for key metabolites from glycolysis, PPP and purine metabolism. The top ten candidate biomarkers are shown in Fig. 1l. Supporting the potential importance of inosine metabolism within glaucomatous RBCs, inosine metabolic intermediates, including inosinic acid (IMP) and inosine diphosphate (IDP), were extremely strong with the area under the curve (AUC) of 0.97 and 0.95, respectively. Hypoxanthine was also strong, with an AUC of 0.84. Notably, the diagnostic power of these erythrocyte metabolites was comparable to that of established ophthalmic parameters such as MD (AUC 0.99), VA (AUC 0.94), pRNFL (AUC 0.94), IOP (AUC 0.89) and C/D ratio (AUC 0.89). These findings underscore the clinical relevance of inosine metabolism in glaucomatous erythrocytes and highlight its potential as a systemic biomarker complementing conventional ocular examinations.

Taken together, using two independent human cohorts and untargeted high-throughput metabolomic profiling, we provide novel evidence that: (1) decreased RBC number is an unrecognized pathogenic feature of patients with glaucoma and is negatively correlated with disease severity; (2) enhanced RBC oxygen delivery capacity is a compensatory adaptive response to decreased RBC number in patients with glaucoma and negatively links with glaucoma severity; and (3) glucose and inosine metabolic reprogramming are strongly associated with changes of RBC function and numbers in glaucoma patients. These findings reveal a metabolite-based diagnostic signature based on reprogramming glucose and purine metabolism. These findings prompted us to further define the role of inosine in erythrocyte generation and function in glaucoma and how these erythroid-related alterations contribute to retinal neurodegeneration (Fig. 1m).

### AMPK enhances PNP activity to reinforce the inosine compensatory mechanism and promote function and metabolism in mature RBCs

**Fig. 2:**
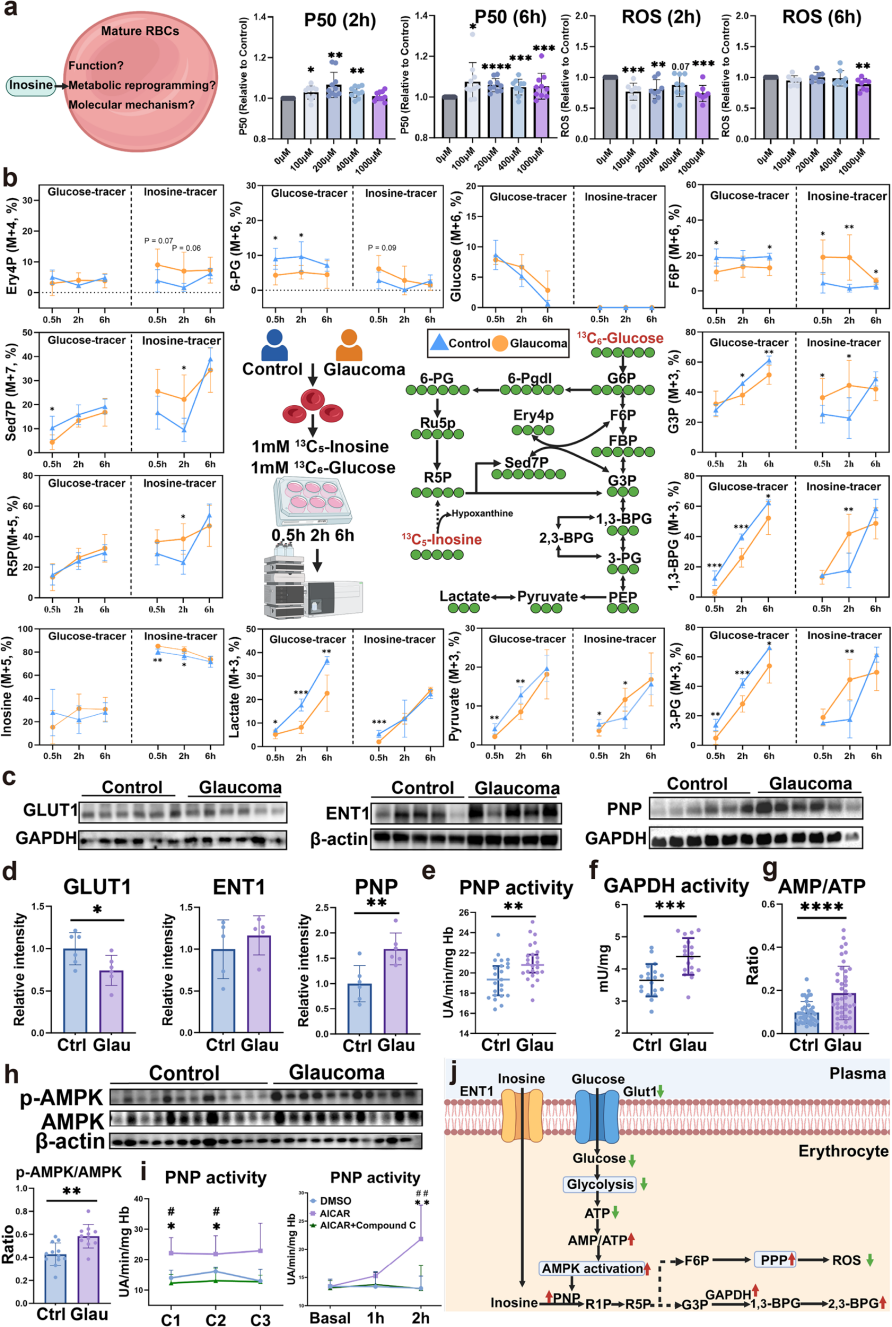
Inosine as an alternative carbon source, promoting erythrocyte function and metabolism. **a**, Scheme illustrating the investigation of the function, metabolic and molecular mechanisms driven by inosine in mature RBCs. The P50 and ROS levels of glaucomatous erythrocytes treated with inosine at different concentrations. **b**, The [^13^C_6_] glucose or [1′,2′,3′,4′,5′-^13^C_5_] inosine tracer in erythrocytes of patients and healthy controls. **c**,**d**, The expression level of GLUT1 and ENT1 in the erythrocyte membrane, and the level of PNP. **c** shows representative Western blot images of GLUT1 and ENT1 expression in the erythrocyte membrane and PNP levels, while **d** presents the corresponding quantitative analysis/statistical summary of these protein levels. **e**–**g**, Comparison of erythrocyte PNP activity (**e**), GAPDH activity (**f**) and the AMP-to-ATP ratio (**g**) between glaucoma (Glau) and controls (Ctrl). **h**, The ratio of phosphorylated AMPK (p-AMPK) to total AMPK in erythrocytes between glaucoma (Glau) and controls (Ctrl). **i**, The PNP activity of erythrocytes treated with AICAR (an AMPK activator) or compound C (dorsomorphin, an AMPK inhibitor) at varying concentrations for 2 h or for different durations at a constant concentration. **j**, Scheme illustrating the low expression of GLUT1 and glucose deprivation activating the energy sensor AMPK, which in turn enhances PNP activity to reinforce the inosine compensatory mechanism for maintaining oxygen release and resisting oxidative stress. Data are presented as mean ± s.d.; *P < 0.05, **P < 0.01, ***P < 0.001.

Glucose is the major fuel to maintain RBC bioenergetics. Our comprehensive untargeted metabolomic profiling revealed that patients with glaucoma exhibit impaired glucose metabolism, featuring decreased glycolysis and PPP but enhanced Rapoport–Luebering shunt (RLS)-induced production of 2,3-BPG. We hypothesize that the observed enhanced inosine catabolism serves to offset the impaired glucose metabolism by functioning as an alternative carbon source to fuel RBC energetics and to promote production of 2,3-BPG and oxygen release. This is a compensatory metabolic adaptation that may represent an intrinsic response to alleviate tissue hypoxia caused by the reduced RBC count observed in patients with glaucoma. To test this hypothesis, we conducted in vitro experiments (from 2 to 6 h) to determine the effects of inosine-mediated oxygen release and anti-oxidative stress capacity in cultured erythrocytes isolated from patients with glaucoma. The results showed that, following inosine treatment, the ability to release oxygen and anti-ROS was enhanced (Fig. 2a). Stratified analysis further revealed that inosine similarly enhanced oxygen release in patients with POAG as well as those with PACG, suggesting that its functional effect is subtype independent (Supplementary Fig. 1d).

Next, we sought to elucidate the specific pattern of metabolic reprogramming of glucose and inosine between the patients with glaucoma and age–sex-matched healthy controls. To do this, we used a stable-isotope-based metabolomics approach to trace the metabolic routes of glucose and inosine, respectively (Fig. 2b). Specifically, we isolated erythrocytes from healthy controls and patients with glaucoma and cultured them in a glucose-free conditioned medium supplemented with either [^13^C_6_] glucose or [1′,2′,3′,4′,5′-^13^C_5_] inosine at the same concentration for different durations (0.5 h, 2 h and 6 h). Both tracers were extensively metabolized via the glycolysis and PPP pathways to produce ^13^C-labeled intermediates. For the ^13^C-labeled glycolytic intermediates derived from ^13^C-glucose, the fractions of ^13^C-labeled metabolites were higher in healthy controls than in patients with glaucoma, including glucose 6-phosphate (G6P), G3P, 1,3-BPG, 3-phosphoglycerate (3-PG), pyruvate and lactate. The fraction of targeted labeled intermediates from glucose in the PPP pathways also showed a decrease in patients with glaucoma, such as 6-PGand sedoheptulose 7-phosphate (Sed7P). By contrast, the ^13^C-labeled intermediates from the [1′,2′,3′,4′,5′-^13^C_5_] inosine tracer were significantly higher in patients with glaucoma than in controls for both glycolysis and PPP pathways (Fig. 2b). For healthy controls, the fraction of labeled intermediates of glycolysis derived from the glucose tracer was higher than that from the inosine tracer, while in patients with glaucoma, the^13^C-labeled fractions were higher from the inosine tracer (Supplementary Table 2). This is consistent with the results from untargeted metabolomics. The flux analyses revealed that, compared with normal erythrocytes, which primarily utilize glucose as a carbon source, glaucomatous erythrocytes have a preference to use inosine as an alternative fuel for the central carbon metabolism (glycolysis and PPP). Thus, metabolomic profiling and isotopic tracing analyses in glaucoma revealed a new but compelling notion that glucose metabolism is impaired in mature erythrocytes of patients with glaucoma, leading to the use of inosine as an alternative carbon source. Inosine-derived R1P is rapidly converted to R5P (a PPP intermediate) and can be metabolized to produce G3P, thereby enhancing glycolysis, which is consistent with the observed changes in 2,3-BPG and the P50 value. However, this compensatory adaptation appears inadequate, as metabolomic data from glaucomatous erythrocytes showed downregulation of the PPP and elevated oxidative stress (Fig. 1j).

Then, we were motivated to delineate the molecular mechanism underlying impaired glucose but enhanced inosine metabolic reprogramming in glaucoma erythrocytes. First, we examined the expression levels of glucose transporter-1 (GLUT1) and ENT1, the primary transporter of inosine. Our findings revealed a significant decrease in GLUT1 expression, while no notable difference in ENT1 was observed on the membranes of glaucoma RBCs (Fig. 2c,d). In addition, we assessed the expression and activity of PNP, a key enzyme in inosine catabolism, and found that both its expression and activity were significantly elevated in patients with glaucoma (Fig. 2d,e). The activity of glyceraldehyde-3-phosphate dehydrogenase (GAPDH) was also enhanced, promoting the synthesis of 1,3-BPG, a substrate for bisphosphoglycerate mutase (BPDM) of the RLS to generate more 2,3-BPG for oxygen release (Fig. 2f). Furthermore, we noted that the ratio of AMP to ATP was significantly higher in patients with glaucoma compared with normal controls (Fig. 2g), indicating a cellular energy disorder in their erythrocytes. Consistently, the AMPK activity quantified accurately by the ratio of phosphorylated AMPK (p-AMPK) to total AMPK was significantly increased in glaucomatous erythrocytes (Fig. 2h). Thus, this finding led to a new possibility that, in glaucoma, the low expression of GLUT1 and glucose deprivation activate the energy sensor AMPK, which in turn enhances PNP activity to promote inosine catabolism as a compensatory mechanism for maintaining energy balance and oxygen release.

To test this hypothesis, we treated the erythrocytes with AICAR (5-aminoimidazole-4-carboxamide ribonucleotide) and compound C (dorsomorphin), a cell-permeable AMPK activator and inhibitor, respectively^33,34^, at varying concentrations (AICAR: 100 μM, 1 mM, 2 mM; AICAR 100 μM with compound C: 10 μM, 20 μM, 40 μM) for 2 h or for different durations at a constant concentration (AICAR at 1 mM and compound C at 20 μM). We found that the AMPK activator, AICAR, induced PNP activity (Fig. 2i), while compound C inhibited AICAR-induced PNP activity. Therefore, we revealed that impaired glucose metabolism induces AMPK activation, enhancing PNP activity in the erythrocytes of patients with glaucoma (Fig. 2j).

### Genetic ablation of murine erythrocyte ENT1 establishes an aging-dependent glaucoma model and reveals a direct role for inosine in erythropoiesis

**Fig. 3:**
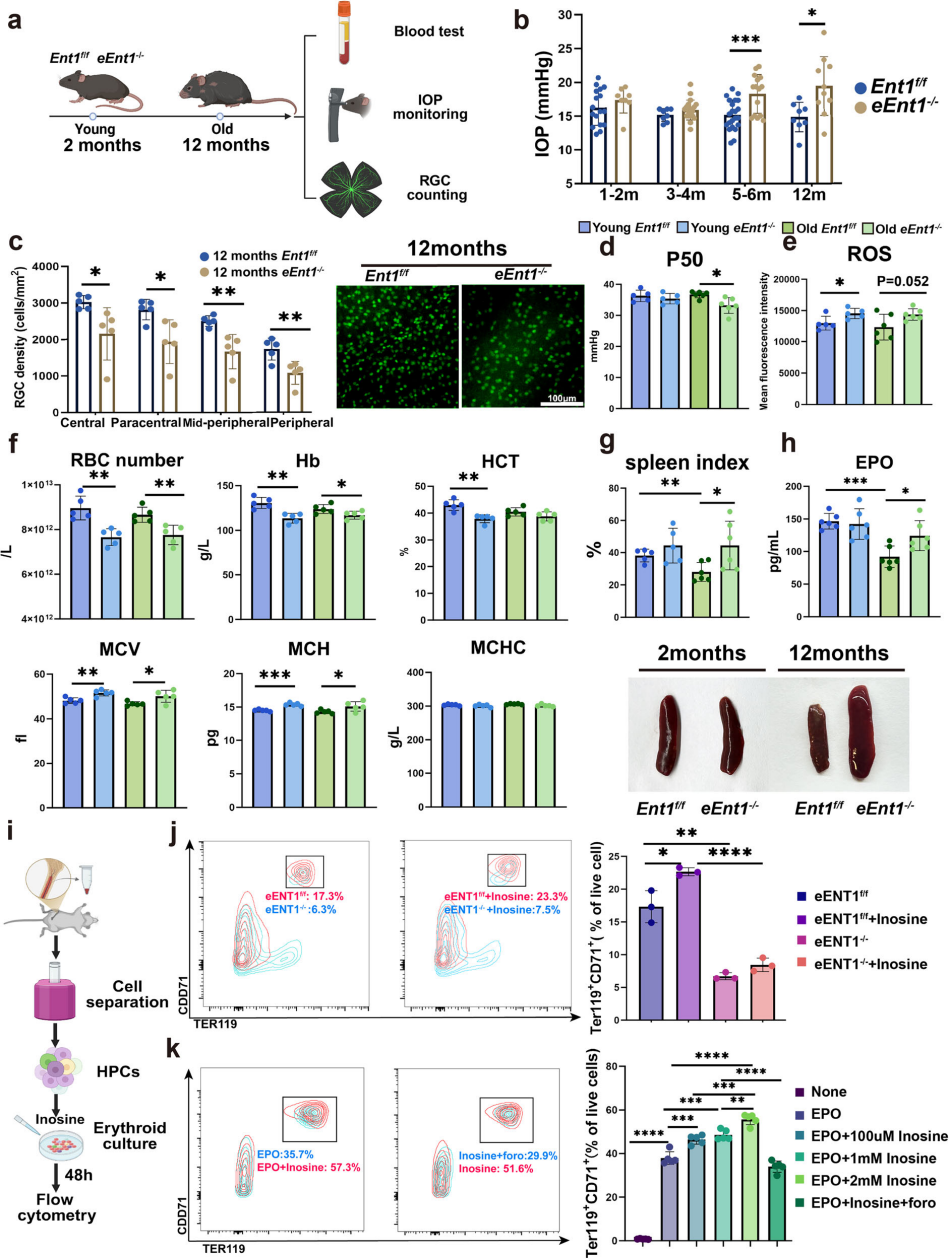
Erythrocyte inosine deficiency establishes an aging-dependent glaucoma model. **a**, Scheme illustrating the comparison of erythrocyte and ocular tests between the young (approximately 2 months old) and aged (over 12 months old) *eEnt1*^*−/−*^ and *Ent1*^*f/f*^ mice. **b**, Comparison of the IOP between the *eEnt1*^*−/−*^ and *Ent1*^*f/f*^ mice at different ages. **c**, RGC density of the aged *eEnt1*^*−/−*^ and *Ent1*^*f/f*^ mice. **d**,**e**, P50 value (**d**) and ROS level (**e**) of the young and aged *eEnt1*^*−/−*^ and *Ent1*^*f/f*^ mice. **f**, Comparison of erythrocyte parameters among the young and aged *eEnt1*^*−/−*^ and *Ent1*^*f/f*^ mice. **g**,**h**, Spleen index (**g**) and EPO levels (**h**) of young and aged *eEnt1*^*−/−*^ and *ENT1*^*f/f*^ mice. **i**, Scheme illustrating the HSPC separation and erythroid culture. **j**,**k**, Comparison of the erythroid lineage differentiation in cultured EPO-stimulated HSPCs isolated from WT, *Ent1*^*f/f*^ and *eEnt1*^*−/−*^ mice with or without inosine and foro (a PNP inhibitor) treatment. **j** shows the comparison of erythroid lineage differentiation in cultured EPO-stimulated HSPCs isolated from *Ent1*^*f/f*^ and *eEnt1*^*−/−*^ mice with or without inosine treatment, while **k** shows the comparison in HSPCs from WT mice with or without inosine and foro (a PNP inhibitor) treatment. Data are presented as mean ± s.d.; *P < 0.05, **P < 0.01, ***P < 0.001.

Given that glaucomatous RBCs preferentially catabolize inosine as an alternative energy source, and that RBCs constitute the most abundant cell type in the human body, this enhanced inosine consumption may deplete systemic inosine availability and disrupt erythroid homeostasis. To investigate whether inosine deficiency contributes to glaucoma progression, we genetically ablated murine erythrocyte ENT1 (Fig. 3a), a major transporter expressed in RBCs of both humans and mice^35^ (Methods and methods). Notably, *eEnt1*^*−/−*^ mice developed age-dependent glaucomatous features: IOP began to increase significantly after 6 months of age and was accompanied by RGC loss in animals over 12 months (Fig. 3b,c), whereas young *eEnt1*^*−/−*^ mice showed no ocular abnormalities. These findings provided proof-of-principle genetic evidence that the absence of ENT1 in erythroid cells predisposes mice to an age-dependent and late-onset glaucoma.

Importantly, the aged *eEnt1*^*−/−*^ mice also exhibited reduced oxygen-releasing capacity (lower P50) and elevated ROS levels in RBCs (Fig. 3d,e), indicating metabolic maladaptation. By contrast, young *eEnt1*^*−/−*^ mice maintained relatively normal RBC oxygen delivery despite similarly reduced RBC count and Hb levels, alongside elevated MCV and MCH (Fig. 3f). This supports a model in which younger mice retain transient compensatory capacity, but cumulative erythropoietic dysfunction and impaired oxygen delivery with advancing age lead to RGC damage and IOP development and progression of glaucoma.

Unexpectedly, we noticed an increased spleen index (spleen weight/body weight × 10) in the aged *eEnt1*^*−/−*^ mice, possibly reflecting a compensatory response to decreased erythropoiesis (Fig. 3g). Consistently, plasma levels of erythropoietin (EPO) were elevated in aged *eEnt1*^*−/−*^ mice (Fig. 3h), ruling out the decreased erythropoiesis in aged *eEnt1*^*−/−*^ mice is due to reduced EPO levels but further strengthening our hypothesis that the deficiency in inosine uptake by ENT1 leads to decreased age-dependent oxygen release and erythropoiesis, which is independent of EPO.

To determine whether inosine directly regulates erythroid differentiation, we cultured HSPCs isolated from WT, *Ent1*^*f/f*^ and *eEnt1*^*−/−*^ mice under EPO-stimulated erythroid differentiation conditions with or without inosine or foro (a PNP inhibitor) (Fig. 3i). The number of Ter119^+^CD71^+^ erythroid cells was significantly diminished in HSPCs from *eEnt1*^*−/−*^ mice after EPO-stimulated culture, which did not respond to inosine (Fig. 3j). The exogenous supplementation of inosine promoted the increase of Ter119^+^CD71^+^ erythroid cells in HSPCs from *Ent1*^*f/f*^ and WT mice; this effect was abolished by cotreatment with foro (Fig. 3j,k). These results provide both genetic and pharmacologic evidence that inosine directly promotes erythropoiesis via the ENT1–PNP axis. Together, these findings establish that erythrocyte-specific ENT1 deletion leads to age-dependent IOP elevation and RGC degeneration, mimicking human glaucoma. Mechanistically, this phenotype is linked to dual failures in erythropoiesis and metabolic adaptation in RBCs due to disrupted inosine uptake and utilization. Our data further suggest that inosine is a critical metabolic regulator of both erythroid regeneration and mature RBC function in the context of glaucomatous neurodegeneration.

### Inosine alleviates glaucoma progression in an age-independent model by improving mature erythrocyte function and erythropoiesis

**Fig. 4:**
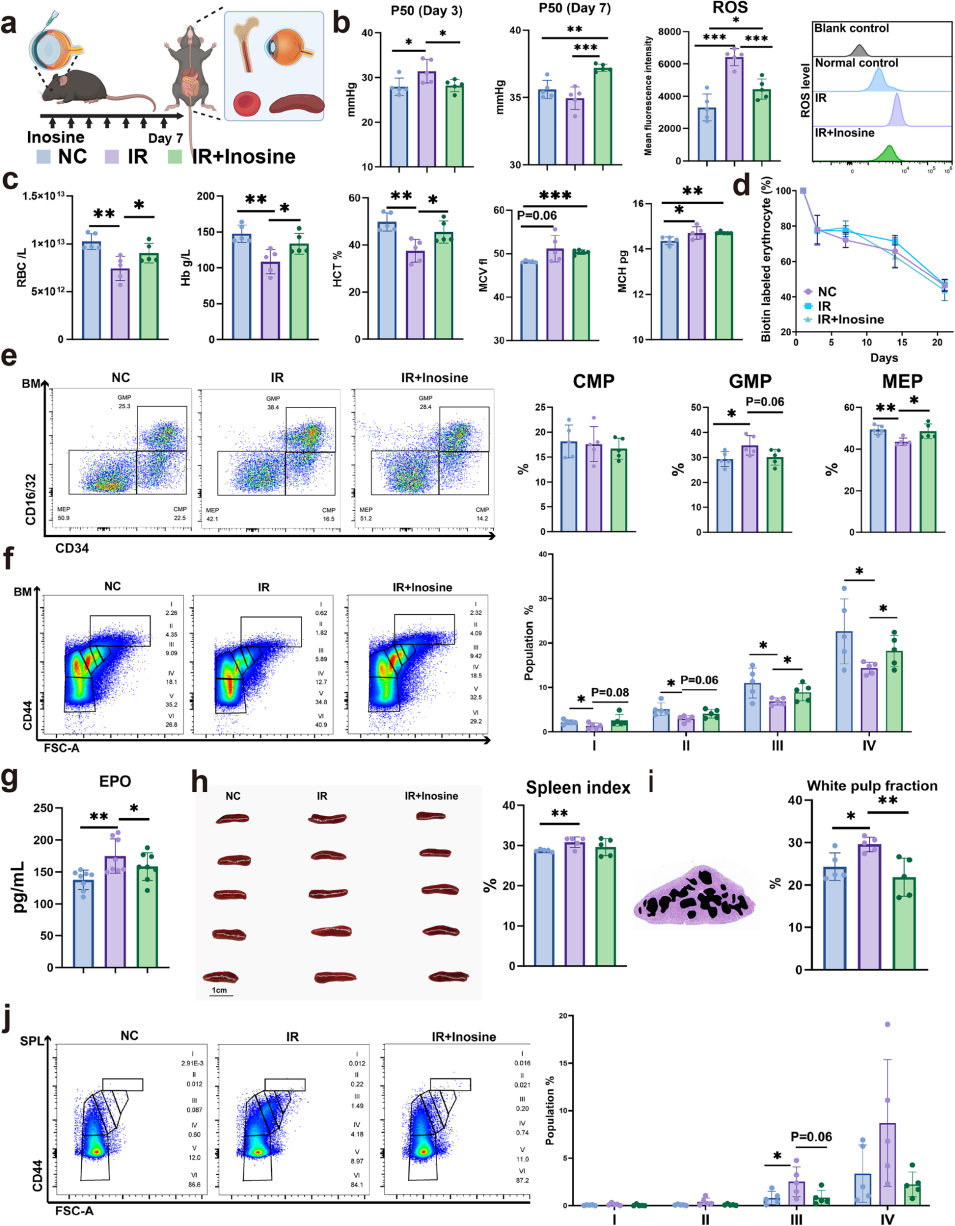
Inosine promotes erythrocyte function and HSC differentiation in vivo. **a**, Scheme illustrating a retinal I/R mouse model of ocular hypertension glaucoma. **b**, P50 and ROS levels in erythrocytes of the normal control group (NC), the saline-treated group after I/R injury, and the inosine-treated group (I/R+inosine) at 3 days and 7 days after I/R injury. **c**, Erythrocyte parameters among the three groups. **d**, Lifespan of circulating erythrocytes. **e**, Flow cytometry analysis of the differentiation of HSCs. HSCs differentiate into CMPs, which further differentiate into GMPs and MEPs. **f**, Terminal differentiation of erythrocytes in bone marrow. **g**, The level of plasma EPO among the three groups. **h**,**i**, Comparison of spleen size, spleen index and the white pulp fraction with H&E staining. **h** shows the comparison of spleen size and spleen index, while **i** presents the comparison of the white pulp fraction of the spleen with H&E staining. **j**, Terminal differentiation of erythrocytes in the spleen among the three groups. Data are presented as mean ± s.d.; *P < 0.05, **P < 0.01, ***P < 0.001.

Utilizing two independent cohorts from Xiangya Hospital and the UK Biobank, we identified that decreased erythrocyte count as a common pathogenic feature among patients with glaucoma. In the above section, we provided genetic evidence that ENT1-mediated uptake of inosine is essential for both RBC generation and function to counteract age-dependent development of glaucoma. To further validate the contribution of ENT1-mediated inosine to RBC function and erythropoiesis in a broader and age-independent context, we took advantage of a retinal I/R mouse model of ocular hypertension. In this model, mice were intraperitoneally injected with inosine daily for 7 days following I/R injury, with saline-treated animals serving as controls (Fig. 4a). Initially, we observed that the P50 value of mature RBCs was elevated 3 days after I/R injury, but subsequently decreased by day 7 (Fig. 4b). Inosine treatment significantly increased the P50 value and reduced ROS levels (Fig. 4b). On the seventh day after I/R injury, RBC count, Hb and HCT were reduced, while MCH was increased in saline-treated mice, resembling clinical hematologic features of patients with glaucoma. Inosine treatment rescued these impairments (Fig. 4c). To exclude the possibility that decreased RBC counts were caused by increased erythrocyte clearance, we assessed erythrocyte lifespan by labeling all circulating erythrocytes in vivo with *N*-hydroxysuccinimidobiotin. Erythrocyte lifespan was unaffected after I/R injury compared with normal controls (Fig. 4d), supporting our conclusion that decreased RBC numbers seen in glaucoma are due to impaired erythropoiesis rather than increased clearance.

Next, we collected bone marrow cells to explore the erythroid progenitor commitment and erythroid differentiation in the I/R model compared with controls. In the bone marrow, hematopoietic stem cells (HSCs) differentiate into common myeloid progenitors (CMPs) and common lymphoid progenitors (CLPs). The CMPs further differentiate into granulocyte–macrophage progenitors (GMPs) and megakaryocyte-erythroid progenitors (MEPs). MEPs give rise to erythroid progenitors, which undergo terminal differentiation to produce mature RBCs^29,36,37^. Compared with the control group (not I/R-treated), the saline- and inosine-treated groups (I/R and I/R + inosine group, respectively) displayed a slight, but insignificant, decrease in the fraction of CMP cells. However, in contrast to the control group, the saline-treated I/R group showed a significant increase in the GMP cells and a decrease in the MEP cells. Compared with the saline-treated I/R group, the inosine-treated I/R group demonstrated a slight decrease in the GMP cells and a significant increase in MEP cells (Fig. 4e). Furthermore, terminal differentiation analysis revealed reduced nucleated erythroblast proportions (I, II, III and IV) in saline-treated I/R mice, compared with those in the control group. By contrast, inosine treatment restored erythroblast proportions to the I/R group (Fig. 4f). To rule out the possibility that the decrease in RBC number is caused by EPO hyposecretion, we also measured the EPO level in plasma after I/R injury, which was found to be compensatorily increased (Fig. 4g). In mice, the spleen is responsible for stress erythropoiesis^38^ when the bone marrow capacity for erythropoiesis is saturated. We assessed the spleen index and observed an increase following I/R injury (Fig. 4h). Hematoxylin and eosin (H&E) staining indicated an expansion of the white pulp and disrupted junctions between red and white pulp following I/R injury (Fig. 4i). Flow cytometry analysis revealed that erythroid precursors in the saline-treated I/R group significantly increased in population III and population IV during the terminal differentiation of erythrocytes (Fig. 4j). The data collectively suggest that the reduction in RBC counts seen in glaucoma is due to the erythroid dysplasia in bone marrow, along with compensatory splenic hematopoiesis.

**Fig. 5:**
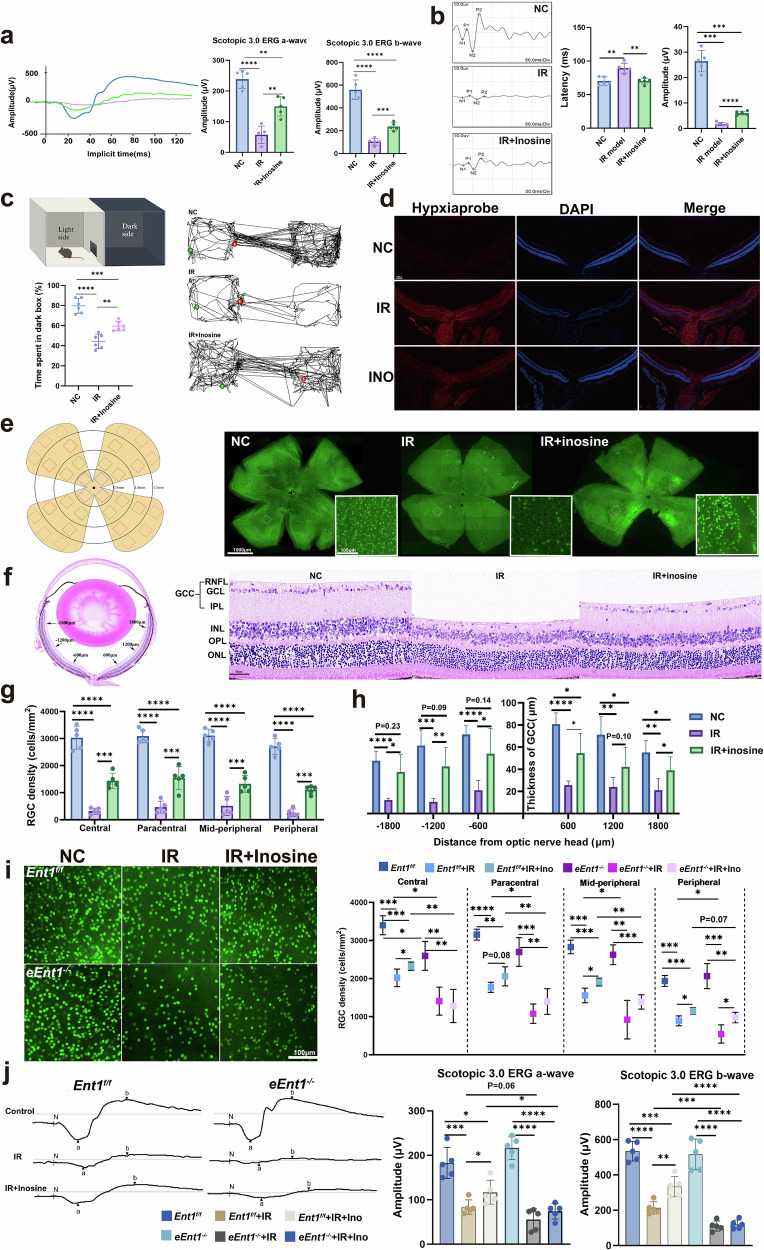
Inosine alleviated RGC loss by promoting mature erythrocyte function and HSC differentiation. **a**–**c**, Visual function was detected using scotopic a-wave and b-wave analysis, VEP testing and a light–dark box test among the normal control group (NC), the saline-treated group after I/R injury, and the inosine-treated group (I/R+inosine). In the light–dark box test, the green dot represents the beginning location of the mice, and the red dot represents the endpoint. **d**, Detection of hypoxic conditions of the retina among the three groups. **e**,**g**, Comparison of the survival of RGCs among these groups. **f**,**h**, Comparison of GCC thickness among the three groups. **f** shows representative images of GCC thickness among the three groups, while **h** presents the corresponding quantitative analysis/statistical summary. **i**,**j**, Comparison of RGC survival, as well as the scotopic a-wave and b-wave analysis, among the young *eEnt1*^*−/−*^ and *Ent1*^*f/f*^ mice after I/R injury with or without inosine treatment. **i** shows RGC survival among young *eEnt1*^*−/−*^ and *Ent1*^*f/f*^ mice after I/R injury with or without inosine treatment, and **j** presents the corresponding scotopic a-wave and b-wave analysis under the same conditions. Data are presented as mean ± s.d.; *P < 0.05, **P < 0.01, ***P < 0.001.

For the visual system, we further assessed visual function by using scotopic a-wave and b-wave analyses (Fig. 5a), a visual evoked potential (VEP) test (Fig. 5b) and a light–dark box test (Fig. 5c). Compared with saline-treated I/R mice, inosine-treated I/R mice exhibited higher VEP amplitudes, increased scotopic a- and b-wave amplitudes and shorter VEP latencies, and spent more time in the dark compartment, indicating improved visual function. As for visual structure, we evaluated retinal hypoxia using a hypoxia probe (Fig. 5d), quantified RGC survival using retinal flat-mounted immunofluorescence (Fig. 5e,g), and assessed retinal structure with H&E staining (Fig. 5f,h). The results demonstrated that inosine treatment improved retinal oxygenation, reduced RGC loss and preserved ganglion cell complex (GCC) thickness in the I/R mice.

Next, to confirm that these effects were mediated through erythrocyte function, we conducted the I/R modeling on young *eEnt1*^*−/−*^ and *Ent1*^*f/f*^ mice. Compared with the *Ent1*^*f/f*^ mice, the *eEnt1*^*−/−*^ mice only exhibited a reduced number of RGCs in the central region before I/R injury, but exhibited more severe RGC loss across all retinal zones after I/R injury (Fig. 5i). After I/R injury with inosine treatment, the *eEnt1*^*−/−*^ mice showed slight preservation of RGCs in the peripheral region. This may be attributed to the direct neuroprotective function of inosine on RGCs (see the following section). A similar trend in visual function was observed in the scotopic a-wave and b-wave among the six groups (Fig. 5j). In summary, our study provides strong evidence that inosine can effectively restore visual structure and function largely by enhancing oxygen release and reducing oxidative stress in mature erythrocytes, and by promoting HSC differentiation toward erythropoiesis.

### Inosine enhances RGC energy metabolism and survival under hypoxia

As both erythroid generation and the metabolic adaptation of mature erythrocytes in patients with glaucoma rely on inosine, and considering that erythrocytes account for the majority of cells in the human body, their heightened utilization of inosine may substantially deplete circulating inosine levels. This systemic reduction could limit inosine availability to other metabolically vulnerable tissues, such as RGCs under glaucoma stress.

**Fig. 6:**
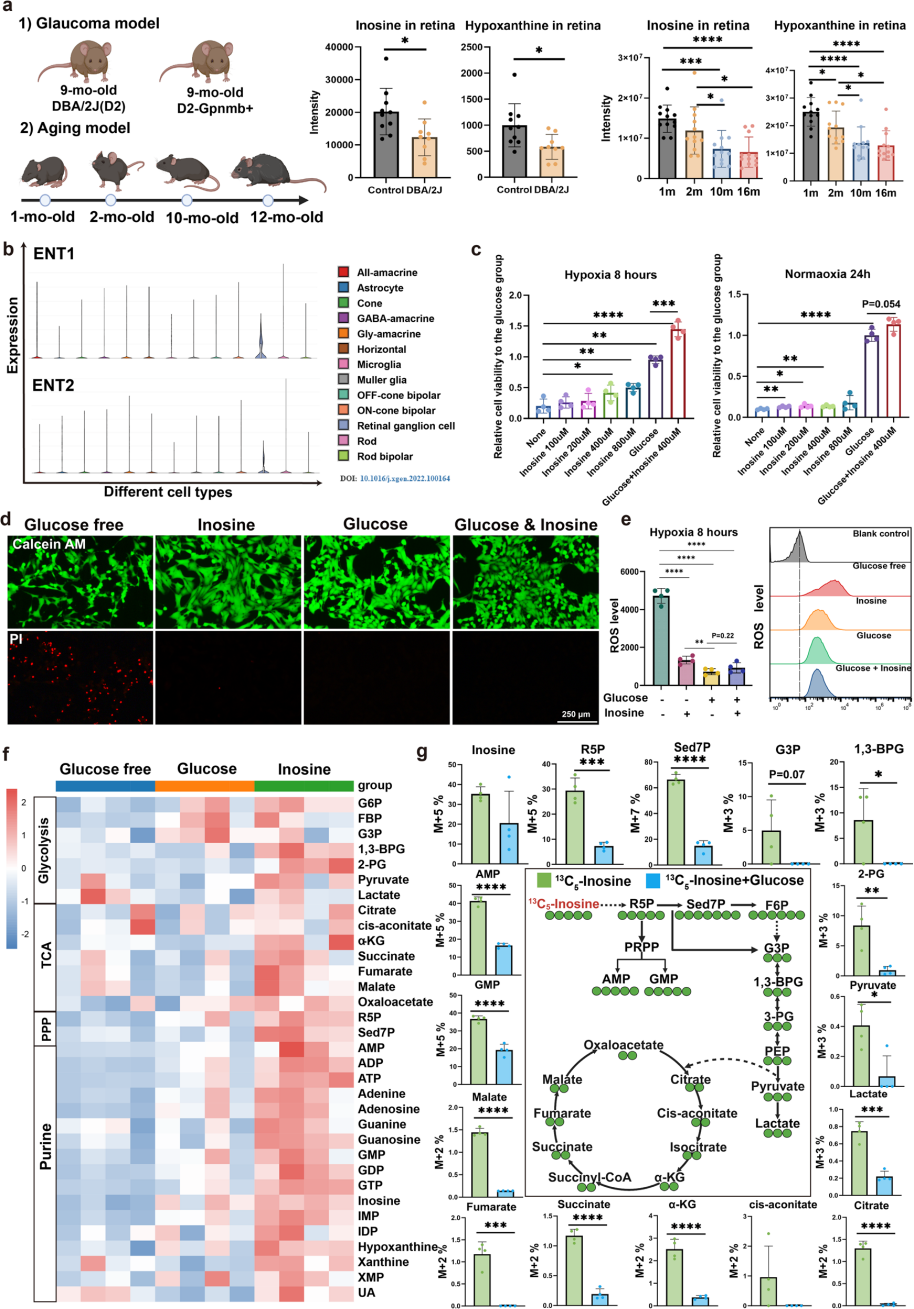
The direct neuroprotective effect of inosine on RGCs. **a**, The level of inosine and hypoxanthine changes in the retina between the DBA/2J mice (D2) and the D2-Gpnmb+ mice (control), or among mice at different ages. **b**, ENT1 and ENT2 expression among different cells of the retina. **c**, Cell viability of RGCs treated with inosine under hypoxic conditions for 8 h and under normoxic conditions for 24 h. **d**, Costaining with calcein-AM (live cells, green) and propidium iodide (dead cells, red) of RGCs under conditions of oxygen and glucose deprivation for 8 h, and treated with glucose or inosine. **e**, ROS level of RGCs under different conditions. **f**, Heatmap of the RGC intermediates of glycolysis, the PPP, the glutathione synthesis pathway and purine metabolism. **g**, The [1′,2′,3′,4′,5′-^13^C_5_] inosine tracer in RGCs with or without glucose under hypoxia for 8 h. Data are presented as mean ± s.d.; *P < 0.05, **P < 0.01, ***P < 0.001.

To explore whether inosine availability is altered during glaucoma progression, we examined metabolomic data from a published dataset on the DBA/2J mouse model of glaucoma^16^, a well-established model characterized by spontaneous age-related IOP elevation and RGC loss. At 9 months of age, when IOP is significantly increased but has not yet developed glaucoma, we found that retinal inosine and its downstream catabolite hypoxanthine were significantly decreased (Fig. 6a). These findings indicate that inosine depletion is an early event in glaucoma pathogenesis and may contribute to retinal metabolic insufficiency before neurodegeneration becomes apparent. Furthermore, given that aging is a major risk factor for glaucoma, we investigated age-related changes in retinal purine metabolism in healthy mice. Strikingly, both inosine and hypoxanthine levels were significantly reduced in the retina with aging alone, even in the absence of elevated IOP (Fig. 6a). This suggests that inosine utilization is susceptible to dysregulation by both IOP elevation and physiological aging—two converging risk factors for glaucoma—which may synergistically impair retinal metabolic resilience. To identify the retinal cell types that might depend on inosine, we analyzed single-cell transcriptomic data from the Single Cell Portal. We found that ENT1 and ENT2—the primary transporters of inosine—are highly expressed in RGCs relative to other retinal cell types (Fig. 6b), suggesting that RGCs may actively utilize inosine. Based on this, we hypothesized that inosine could serve as a metabolic substrate for RGCs to combat glaucoma stress, such as hypoxia, redox imbalance and energy failure. To test this, we cultured R28 retinal cells under normoxia and hypoxia, with or without glucose or inosine supplementation. Cell viability assays (CCK-8) showed that inosine promoted RGC survival in a dose-dependent manner under glucose-free conditions, regardless of oxygen availability. Notably, cotreatment with inosine and glucose yielded significantly higher viability than glucose alone (Fig. 6c), suggesting that inosine provides additive metabolic support. To further confirm the ability of inosine to resist hypoxia, the RGCs were cultured under conditions of oxygen and glucose deprivation for 8 h and treated with glucose or inosine, or both. The costaining with calcein-AM (live cells, green) and propidium iodide (dead cells, red) indicated that inosine protected the RGCs from death under hypoxic and glucose-free conditions (Fig. 6d). Moreover, we also investigated the levels of ROS in these groups and found that RGCs without oxygen and glucose exhibited a higher oxidative stress level, which was reduced by inosine or glucose treatment (Fig. 6e).

To delineate the metabolic mechanisms, we performed untargeted metabolomics in RGCs cultured under glucose-free hypoxia. Inosine treatment significantly activated glycolysis, the PPP and purine metabolism, leading to increased intracellular ATP and GTP levels (Fig. 6f). We further conducted ^13^C-tracer experiments using uniformly labeled [1′,2′,3′,4′,5′-^13^C_5_] inosine to map its metabolic fate. The ^13^C label was incorporated into intermediates of glycolysis, the PPP and the tricarboxylic acid (TCA) cycle—especially under glucose-free conditions, where the labeling fraction was higher than in glucose-treated cultures (Fig. 6g). Collectively, our findings reveal that inosine serves as a critical fuel required by three key cell populations affected in glaucoma: mature erythrocytes, erythroid progenitors and RGCs.

## Discussion

**Fig. 7:**
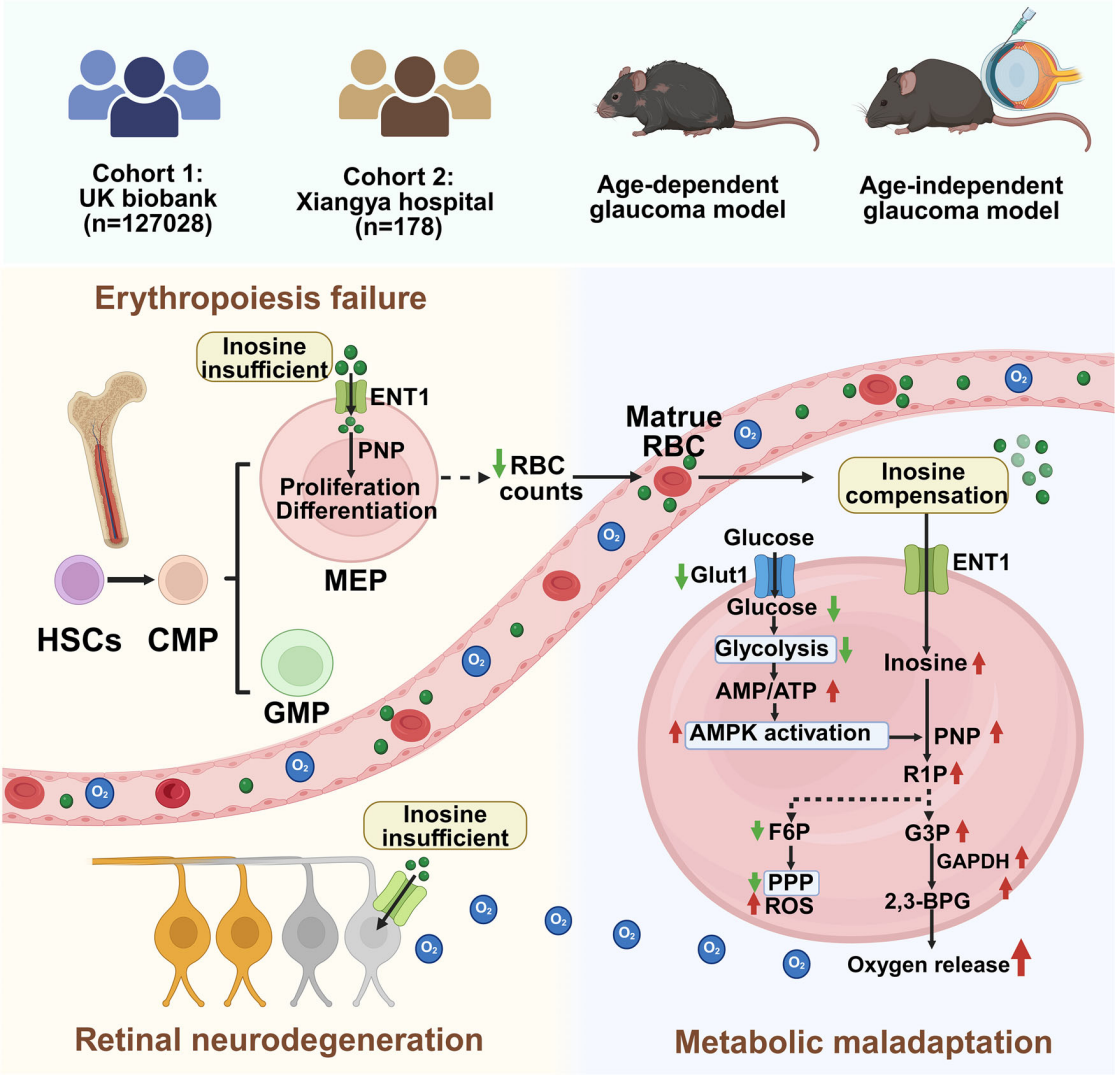
Scheme illustrating the key findings in this study. A graphical summary of experimental findings across two human cohorts and age-dependent and age-independent glaucoma animal models reveals a multitiered pathophysiological axis linking bone marrow erythropoiesis, mature erythrocyte metabolic maladaptation and retinal neurodegeneration in glaucoma. In the bone marrow (left), inosine insufficiency impairs ENT1/PNP-mediated erythroid proliferation and differentiation from MEPs, leading to impaired erythropoiesis and reduced RBC count. In mature RBCs (right), reduced glucose uptake due to GLUT1 reduction suppresses glycolysis and the PPP, resulting in ATP depletion and oxidative stress. To compensate for retinal hypoxia and reduced RBC number, mature RBCs shift toward AMPK-driven inosine catabolism via PNP, generating R1P, G3P and 2,3-BPG to enhance oxygen release. However, excessive inosine utilization by abundant RBCs depletes the circulating inosine, limiting its availability to erythroid precursors and RGCs. This systemic inosine competition contributes to retinal hypoxia and neurodegeneration (bottom). These findings redefine glaucoma as a systemic erythroid–retinal metabolic disorder and highlight inosine metabolism as a therapeutic target.

Before the research reported here, nothing was known about erythrocyte generation and their functional changes in glaucoma. Here, we define the changes in the number, function and metabolism of erythrocytes, the underlying mechanism and their specific contributions to glaucoma. We further identify multiple novel metabolite-based diagnoses and describe possible inosine-based therapies for glaucoma that promote RBC oxygen delivery, RBC generation and RGC survival to combat the progression of glaucoma. Our study supports a new but compelling concept that glaucoma is not merely a localized ocular disease but rather an erythroid-linked metabolic syndrome: Under glaucomatous conditions, metabolic reprogramming in mature RBCs and impaired erythropoiesis increase dependency on inosine to support oxygen delivery and erythropoiesis. Simultaneously, RGCs rely on inosine to maintain energy homeostasis and redox balance under hypoxic stress. Realizing that erythrocytes constitute over 80% of all cells in the human body, their overwhelming demand for inosine creates a systemic competitive environment, depleting the circulating inosine and limiting its availability to RGCs and erythropoietic progenitors. Thus, compensatory increased oxygen delivery from mature RBCs mediated by enhanced inosine uptake and catabolism is insufficient to counteract decreased erythropoiesis-induced hypoxia and retinal neurodegeneration. Instead, this intercellular competition for inosine may underlie a pathological feedforward loop in which defective erythropoiesis and retinal neurodegeneration reinforce one another. These observations underscore the biological rationale and therapeutic potential of exogenous inosine supplementation to restore metabolic homeostasis, support erythropoiesis and protect RGCs in glaucoma (Fig. 7).

Initially, we identified a consistent reduction in erythrocyte counts among glaucoma patients using both the UK Biobank (*n* = 127,028) and an independent hospital cohort from Xiangya Hospital (*n* = 178), with results adjusted for age and sex. These findings overcome long-standing inconsistencies in the alteration of RBC count, RBCs, Hb, HCT, MCV and MCH in glaucoma. Although impaired erythrocyte hemorheology has been linked to glaucoma progression^39,40^, previous studies have reported contradictory trends in erythrocyte parameters. These studies may be subject to several limitations, including small sample sizes^39,41^, as well as the lack of 1:1 age and sex matching^42–44^. In addition, by rigorously adjusting for age and sex, our data conclusively link erythropoietic insufficiency and impaired erythrocyte function with disease prevalence and severity, as reflected by longer disease duration, worse visual field deficits and thinner retinal nerve fiber layers. This systemic hematological signature provides a new perspective on the progression of glaucoma and indicates that impaired erythropoiesis is an important systemic contributor to disease progression.

In response to reduced erythropoiesis and retinal hypoxia, we observed a compensatory metabolic reprogramming in mature erythrocytes aimed at enhancing oxygen release, as reflected by elevated P50 values and upregulation of 2,3-BPG. However, this adaptation occurs alongside signs of intracellular glucose deprivation, indicated by reduced GLUT1 expression and suppressed glycolysis and PPP activity. This metabolic suppression leads to ATP depletion and increased ROS accumulation, impairing erythrocyte integrity and function^45,46^. Interestingly, we observed selective accumulation of glycolytic intermediates, G3P and 2,3-BPG, prompting investigation into alternative carbon sources. Metabolomic and flux analyses revealed enhanced inosine catabolism in glaucomatous erythrocytes, indicated by the decrease of inosine, as well as the increase of both hypoxanthine, R1P and PNP activity. R5P, produced from R1P^47^, can be metabolized to produce G3P, which can further generate F6P and 2,3-BPG, thereby activating both glycolysis and PPP. However, this inosine-driven compensation was ultimately inadequate: in glaucomatous erythrocytes without inosine supplementation, the PPP remained suppressed with elevated ROS levels, and ATP concentrations were significantly depleted. In addition, both plasma and intracellular inosine levels declined with disease progression, suggesting a gradual exhaustion of this adaptive mechanism. Moreover, we investigated how the inosine compensatory mechanism of erythrocytes in glaucoma is initiated. In addition to the remarkable decrease in ATP in glaucomatous erythrocytes, we observed a significantly increased AMP-to-ATP ratio, indicating an energy imbalance that could activate AMPK, a key cellular energy sensor. A previous proteomic study identified PNP as a potential substrate of AMPK in RBCs; however, this potential interaction was not experimentally validated^48^. In the present study, we provide the first direct evidence that the enhanced inosine catabolism is triggered by AMPK-mediated activation of PNP, representing a novel regulatory axis, AMPK–PNP–inosine signaling, in RBC metabolism. Altogether, glaucomatous erythrocytes undergo AMPK-driven inosine catabolism to compensate for erythropoietic dysfunction and sustain oxygen release, but this adaptation ultimately fails due to persistent PPP suppression, ATP depletion and declining inosine availability. While our biochemical and pharmacologic data strongly support an AMPK-dependent regulation of PNP, further genetic validation in erythroid cell-specific AMPK or PNP conditional knockout models will be essential to establish definitive causality. Importantly, from metabolomic and flux analyses, we also discovered that erythrocytes in patients with glaucoma preferentially uptake and utilize inosine. Given that erythrocytes constitute approximately 83% of all human cells, this dominant consumption markedly depletes systemic inosine pools. Consequently, inosine availability to other metabolically demanding cells—particularly erythroid progenitors and RGCs—is reduced. This intercellular metabolic competition may exacerbate erythropoietic failure and diminish RGC resilience under hypoxic conditions, forming a metabolic bottleneck that links hematological dysfunction to neurodegeneration. To probe causality, we conducted the genetic ablation of murine erythroid ENT1. Unexpectedly, the *eEnt1*^*−/−*^ mice displayed impaired erythropoiesis early in life but a normal P50 level. However, as they aged, they spontaneously developed progressive systemic oxygen transport failure, elevated IOP and eventual RGC loss, recapitulating the key spectrum of glaucomatous features. These results confirm that erythrocytes not only govern peripheral oxygen delivery but also play a previously unrecognized role in retinal metabolic homeostasis. In this model, dyserythropoiesis and compensatory adaptation of mature RBCs precede glaucoma development. Despite normal EPO levels, *eEnt1*^*−/−*^ mice exhibited suppressed erythroid differentiation, increased splenic compensatory erythropoiesis and elevated ROS levels, also mirroring clinical observations in patients with glaucoma.

Mechanistically, erythroid progenitors from *eEnt1*^*−/−*^ mice failed to respond to exogenous inosine in vitro, and inhibition of PNP abrogated inosine’s pro-erythropoietic effects in WT mice and *Ent1*^*f/f*^ mice. These results confirm that inosine promotes erythropoiesis via the ENT1–PNP axis and is essential for maintaining systemic oxygen delivery. Moreover, the age-related increase in IOP and RGC degeneration observed in *eEnt1*^*−/−*^ mice may be partially explained by the impaired ability of erythroid cells to clear circulating adenosine and inosine. Under physiological conditions, RBCs rapidly remove adenosine from circulation, absorbing approximately 54% within 1 min via ENT1^35^. The elevated IOP observed in erythroid ENT1-deficient mice is probably multifactorial. Hypoxia-induced ECM-remodeling and profibrotic responses in trabecular meshwork cells can increase outflow resistance^49^. In addition, metabolomic analysis of DBA/2J glaucoma mice and physiological aging mice revealed that retinal inosine and its catabolite hypoxanthine progressively decline with both elevated IOP and aging, before overt neurodegeneration. The consequent mechanical and metabolic stress on the retina, combined with systemic oxygen delivery deficits, culminates in progressive RGC loss in aging *eEnt1*^*−/−*^ mice.

Beyond the hematopoietic system, we demonstrate that inosine also acts directly on RGCs. Public single-cell datasets reveal high ENT1 and ENT2 expression in RGCs, and functional experiments show that inosine supports RGC survival under glucose deprivation and hypoxic stress by fueling glycolysis, PPP and TCA cycle intermediates. Isotope tracing further confirms that inosine-derived carbon is integrated into key metabolic pathways within RGCs. These findings position inosine as both a systemic and neuron-specific metabolic protector in glaucoma. So far, no studies have explored the use of inosine as a neuroprotective strategy in glaucoma. While early research in this century explored the role of inosine in promoting the regeneration of RGC axons, these studies primarily focused on lower vertebrates, which possess regenerative capacity following optic nerve injury^50^ and did not investigate the precise mechanisms involved^51^, nor did they explore glaucoma animal and cell models or conditions of hypoxia.

Finally, we validated the therapeutic potential of inosine using an ocular hypertension model of glaucoma. Inosine administration restored erythropoiesis, enhanced oxygen-releasing capacity of mature erythrocytes, reduced oxidative stress, and preserved both retinal structure and visual function. It also rescued erythroid progenitor commitment in vivo. Notably, the protective effects of inosine were blunted in erythroid-specific ENT1-deficient mice. In particular, we observed that young *eEnt1*^*−/−*^ mice subjected to I/R injury exhibited worse visual function and retinal structure compared to *Ent1*^*f/f*^ mice after I/R injury, underscoring that the majority of inosine’s therapeutic efficacy relies on erythrocyte-mediated mechanisms. Together, these findings establish inosine as a multitarget therapeutic agent that operates at the interface of erythropoiesis, erythrocyte function and retinal neuroprotection.

Growing evidence underscores the importance of restoring metabolic and redox homeostasis in both the retina and systemic compartments as a promising therapeutic avenue for glaucoma. Previous studies have shown that NAD supplementation may enhance mitochondrial redox capacity and delay visual field loss^52^, while pyruvate can restore energy balance and protect RGCs by bypassing impaired glycolytic steps^16,53^. In this context, inosine emerges as a compelling dual-action metabolic substrate: it can fuel glycolysis, PPP and the TCA cycle in both erythroid and neuronal cells, thereby supporting ATP production and antioxidant defense. Importantly, inosine not only promotes erythropoiesis and enhances the oxygen-releasing capacity of mature erythrocytes under stress but also directly supports RGC survival under hypoxic and glucose-deprived conditions. By alleviating systemic inosine depletion and improving metabolic resilience in multiple cell types simultaneously, inosine may outperform single-target metabolic interventions. These dual effects position inosine as a metabolically versatile candidate for the treatment of glaucoma, capable of addressing both the oxygen transport deficit and retinal energy failure that underlie neurodegeneration. Furthermore, while inosine shows therapeutic promise, its long-term safety, optimal dosing and tissue-targeted delivery strategies require rigorous preclinical and clinical evaluation.

In conclusion, our findings redefine glaucoma as a systemic erythroid–retinal metabolic disorder driven by erythropoietic failure and inosine deficiency. Consistent with the concept that erythrocytes act as a metabolically active circulating organ integrating systemic energy and redox regulation^54^, we propose a pathophysiological model in which excessive inosine consumption by abundant RBCs, to combat the reduction in RBC count and retinal hypoxia, leads to systemic inosine depletion, impairing both erythroid regeneration and RGC survival. The resulting metabolic competition between erythroid and neuronal cells exacerbates retinal hypoxia and accelerates neurodegeneration. By targeting inosine metabolism, we uncovered new diagnostic biomarkers and therapeutic strategies for glaucoma, paving the way for metabolically-informed clinical management of this complex neurodegenerative disease.

## Supplementary information

Supplementary Information

## Data Availability

All data generated for this study are available from the corresponding authors upon reasonable request.
