# Supplementary material for: Erythropoiesis–inosine metabolic axis failure underlying retinal neurodegeneration in glaucoma: novel diagnoses and therapies
Source: Exp Mol Med. 2026 Feb 13;58(2):562–78. doi: 10.1038/s12276-026-01654-x (PMC12992561; doi:10.1038/s12276-026-01654-x)
Supplement: Supplementary file 1 — Supplementary Information [file 12276_2026_1654_MOESM1_ESM.pdf]

Supplementary Materials

Supplementary Methods

Supplementary Table 1. List of the significant differential metabolites in erythrocytes between glaucoma patients and control individuals

Supplementary Table 2. The [$^{13}C_6$] glucose and [1′,2′,3′,4′,5′-$^{13}C_5$] inosine tracer in erythrocytes of patients and healthy controls

Supplementary Figure 1. The changes in the number, function, and metabolism of erythrocytes among different subtypes of glaucoma

Supplementary Methods

UK Biobank database

Data from the UK Biobank were obtained following the approval of a valid application through https://www.ukbiobank.ac.uk. The UK Biobank received ethical approval from the North West Multi-Center Research Ethics Committee (approval number: 11/NW/03820). Written informed consent was collected from 502,142 participants. During the baseline recruitment visit, participants completed a self-administered questionnaire covering sociodemographic information, including age, sex, BMI, and disease history. According to the self-report, a total of 180,395 individuals from the UK Biobank with eye problems were included in this project. After excluding participants with other ocular diseases, such as diabetes-related eye disease, injury resulting in loss of vision, macular degeneration, and other serious eye conditions, and individuals without CBC test results, 7138 individuals diagnosed with glaucoma and 119,710 individuals with none of the above diseases as controls were ultimately selected.

Erythrocyte ROS detection

The ROS level of erythrocytes was measured by flow cytometry with the ROS Assay Kit (BL714a, Biosharp). Briefly, 1μL of RBC pellet was incubated with the diluted H2DCFH-DA (1:3000 in PBS) at 37℃ for 30 minutes away from light. After 3 times washing with PBS, the samples were resuspended in 100μL PBS and analyzed by a BD FACS Celesta cytometer.

Retinal flat-mounted immunofluorescence

The eyeballs were fixed with 4% paraformaldehyde for 1 hour at room temperature,

followed by retinal dissection. After the removal of the cornea, lens, and sclera, the retina was cut into a four-leaf clover and incubated in 2% BSA dissolved in PBS containing 0.5% Triton-X 100 for 1 hour. The retina was stained with an anti-Brn3a antibody (Abcam, ab245230 1:100) or anti-Iba1 antibody (Abcam, ab48004 1:200) with an Anti-MHC Class II antibody (Abcam, ab23990 1:200), overnight at 4℃ at least for 16 hours. On the next day, the retina was washed with 0.5% Triton-X10 three times for 5 minutes and fixed in the 4% paraformaldehyde for 10 minutes. Then the retina was incubated with their secondary antibody at room temperature for 2 hours. After that, the retina was washed with PBS, mounted, and photographed by using a Nikon Ni-U microscope (Nikon, Tokyo, Japan). Brn3a-positive, Iba1-positive, and MHCII-positive cells were counted by ImageJ to determine the RGC and microglia density.

Flash electroretinogram (FERG) detection

After 12 hours of dark adaptation, anesthesia and pupil dilation, the mice were positioned on the operating table, and carboxymethyl cellulose gel was applied to maintain corneal moisture. All experimental procedures were performed in the dark, illuminated only by a dim red light. Two reference electrodes were subcutaneously implanted on either side of the nose, while a ground electrode was placed on the back of the mouse. Two recording electrodes, in the form of rings, were positioned in contact with the bilateral corneas. The amplitudes of a-wave and b-wave were detected and measured by Roland Consult.

Flash visual-evoked potential analysis

After 15 minutes of dark adaptation, mice were anesthetized, and three electrodes were inserted under the skin of the mouse's back (ground electrode), anterior bregma (cathode), and occipital bone (anode) respectively. The contralateral eye was covered and the visual function was assessed and recorded using a multifocal electroretinography recorder (GT-2008V-VI, Gotec, Chongqing, China) and Ganzfeld electrodiagnostic system (Gotec).

Light-dark box test

At the start of the test, mice were placed in the light compartment of the light-dark box. Over nine minutes, they were allowed to explore the light and dark compartments freely, and their behavior was recorded.

Histology

The eyeball, kidney, and spleen were fixed in 4% paraformaldehyde and embedded in paraffin for sectioning. Hematoxylin and eosin (H&E) staining was performed, and the slides were examined under light microscopy. The retinal ganglion cell complex thickness (GCC) was assessed at 600, 1200, and 1800 μm from the optic nerve center. Renal injury was determined by evaluating glomeruli, tubules, and tubulointerstitial lesions. Tubulointerstitial lesions were assessed based on Tubule dilatation, tubule atrophy, tubule vacuolar degeneration, red cell tubule type, protein tubule type, interstitial edema, and interstitial cell infiltration. The injury score was estimated on a scale of 0–3: 0 indicated no tubular injury, 1 represented 25% injury, 2 represented 25%–50% injury, and 3 represented 51%–100% injury. The average scores observed in ten randomized microscopic fields determined the final score of each sample. For

the H&E staining of the spleen, the size of the white pulp was measured, and the white pulp fraction between different groups was compared.

Tissue Hypoxia Detection

The level of retinal hypoxia was detected by using the Hypoxyprobe kit (Hypoxyprobe Omni Kit, Hypoxyprobe, Inc.), as previously detailed[22.] Mice were administered with pimonidazole (50 mg/kg body weight) intraperitoneally for 1 hour before being anesthetized. Following transcardial perfusion, the eyes were dissected, fixed, and embedded in paraffin for sectioning. The sections were then subjected to immunohistochemical staining using a rabbit anti-pimonidazole antibody (PAb2627AP, 1:100) and an Alexa Fluor 594-conjugated goat anti-rabbit antibody (A-11037, 1:1000, Thermo Fisher).

ELISA

After coating the wells overnight and washing them, 200 μL of blocking buffer was added to each well and incubated at 37°C for 1 hour. Samples were then added to the appropriate wells and incubated at 37°C for 2 hours. Next, 100 μL of the diluted detection antibody working solution was added to each well and incubated at 37°C for 1 hour. The process was followed by incubation with enzyme conjugates and the chromogenic substrate, which were added sequentially. Following the stop reaction, the plate was read at 450 nm, and the optical density (O.D.) of each well was measured.

Cell culture

Rat retinal precursor (R28) cells, a retinal precursor cell line, were widely utilized to investigate the pathological mechanism of RGC death and the neuroprotection of some therapy *in vitro*. R28 cells were cultured in d in Dulbecco's modified Eagle's medium (DMEM) (Procell, Wuhan, China) supplemented with 10% FBS (Gibco, Grand Island, NY, USA) at 37°C with 5% CO2. The R28 cells in a 10 cm culture dish were digested, centrifuged, and resuspended in 1 mL of culture medium, and mixed well by pipetting. Then, take 10 ul and drop it into the cell counting chamber, count the cells, and then seed them into a 12-well plate equally, with a total volume of 1 mL of culture medium in each well. Cultivate them in a cell culture incubator for 24 hours. Then, the original culture medium was discarded and a glucose-free medium and the corresponding concentration of drugs were added. For the hypoxia model, the R28 was placed in an anaerobic incubator (37°C, 94% N2, 5% CO2, 1% O2) for 8 hours and then was performed with CCK8 and Calcein/PI live-dead cell staining.

Supplementary Table 1. List of the significant differential metabolites in erythrocytes between glaucoma patients and control individuals

Compound	FC	log2(FC)	P value
Phosphate	0.41806	-1.2582	3.28E-24
Fumarate	0.65212	-0.61679	2.73E-20
IDP	0.28235	-1.8244	4.10E-20
Malate	0.61452	-0.70247	9.05E-20
Indole-3-acetaldehyde	0.30329	-1.7212	1.44E-19
D-Erythrose 4-phosphate	0.52665	-0.92507	2.46E-19
acyl-C10:1 (O-Decenoyl-L-carnitine)	0.28415	-1.8153	3.51E-19
IMP	0.20082	-2.316	2.10E-17
acyl-C18:2-OH	0.23084	-2.115	2.61E-16
Diphosphate	0.15863	-2.6563	3.38E-16
acyl-C12:1 (O-dodecenoyl-carnitine)	0.35018	-1.5138	1.91E-14
(5Z-8Z-11Z-14Z)-Icosatetraenoic acid	30.323	4.9223	2.83E-14
acyl-C10 (O-Decanoyl-L-carnitine)	0.26846	-1.8972	3.95E-14
Hexadecanoic acid	0.7302	-0.45365	5.07E-13
Pyridoxal	0.80484	-0.31323	7.32E-13
(9Z)-Tetradecenoic acid	0.31555	-1.6641	1.44E-12
Linoleate	0.51777	-0.9496	1.65E-12
acyl-C12 (O-dodecanoyl-carnitine)	0.43904	-1.1876	3.12E-12
UDP-glucose	0.67063	-0.57642	4.85E-12
ADP	0.59528	-0.74836	6.22E-12
ATP	0.5169	-0.95204	8.55E-12
Citrate	0.49756	-1.0071	1.49E-11
(9Z)-Octadecenoic acid	0.5061	-0.9825	2.38E-11
L-Tryptophan	0.79304	-0.33453	3.12E-11
XMP	0.36349	-1.46	3.44E-11
(9Z)-Hexadecenoic acid	0.41846	-1.2568	1.27E-10
Dehydroascorbate	0.53736	-0.89604	1.60E-10
4-Pyridoxate	0.63071	-0.66496	1.78E-10
Anthranilate	0.86263	-0.21319	2.34E-10
6-Phospho-D-gluconate	0.52286	-0.9355	3.58E-10
acyl-C14:1 (Tetradecenoyl Carnitine)	0.4575	-1.1282	5.54E-10
(8Z-11Z-14Z)-Icosatrienoic acid	0.44696	-1.1618	5.72E-10
7alpha-Hydroxy-3-oxo-4-cholestenoate	0.49534	-1.0135	7.41E-10
Prostaglandin G2	1.0445	0.062846	7.58E-10
Taurine	0.39149	-1.353	1.24E-09
2-Oxoglutaramate	0.74892	-0.41711	1.42E-09
(4Z-7Z-10Z-13Z-16Z-19Z)-Docosahexaenoic acid	0.36949	-1.4364	1.69E-09
Succinate	0.75538	-0.40473	3.63E-09
13(S)-HODE	0.23553	-2.086	6.65E-09
9(S)-HODE	0.23553	-2.086	6.65E-09
GTP	0.32848	-1.6061	1.23E-08

Guanine	0.58092	-0.78358	2.04E-08
Dopamine	0.86006	-0.21748	2.63E-08
Tetradecanoic acid	0.53103	-0.91313	3.83E-08
7-10-13-16-Docosatetraenoic acid	0.36817	-1.4416	4.57E-08
Uracil	0.73067	-0.45272	1.33E-07
acyl-C3 (propionyl-carnitine)	0.61824	-0.69375	1.43E-07
Phosphoenolpyruvate	0.57519	-0.79789	1.98E-07
Ornithine	1.2643	0.33839	2.21E-07
Nicotinamide	0.78184	-0.35505	2.75E-07
(7Z-10Z-13Z-16Z-19Z)-Docosa-7-10-13-16-19-pentaenoic acid	0.43443	-1.2028	3.53E-07
L-Tyrosine	1.3861	0.47108	4.09E-07
(9Z-12Z-15Z)-Octadecatrienoic acid	0.48193	-1.0531	6.45E-07
6-Phosphonoglucono-D-lactone	0.4571	-1.1294	8.79E-07
Indoxyl	0.48758	-1.0363	9.05E-07
Undecanoic acid	0.95943	-0.05975	1.43E-06
acyl-C16:1 (Hexadecenoyl-carnitine)	0.67302	-0.57128	1.97E-06
Prostaglandin A2	0.73054	-0.45296	2.60E-06
Phosphocreatine	0.54515	-0.87527	2.87E-06
Prostaglandin D2	0.8192	-0.28771	5.28E-06
Prostaglandin E2	0.8192	-0.28771	5.28E-06
Cadaverine	5.9095	2.563	6.54E-06
2-Phospho-D-glycerate	0.65768	-0.60454	6.61E-06
ADP-D-ribose	1.5673	0.64828	9.13E-06
acyl-C18:2 (Linoleoyl-CoA)	0.80025	-0.32148	1.02E-05
GDP	0.49132	-1.0253	1.08E-05
N-Succinyl-L-glutamate 5-semialdehyde	0.62977	-0.66709	1.49E-05
acyl-C18:1 (O-octadecenoyl-L-carnitine)	0.82123	-0.28413	1.56E-05
L-Arginine	1.7969	0.84549	1.72E-05
N-Acetylneuraminate	0.63604	-0.65282	3.46E-05
acyl-C2 (acetyl-carnitine)	0.80356	-0.31552	3.54E-05
Pyruvate	0.72971	-0.45461	5.13E-05
Spermidine	0.78774	-0.3442	8.74E-05
3-Phospho-D-glyceroyl phosphate	0.48957	-1.0304	9.73E-05
Glutathione	0.82069	-0.28509	0.000109
Hypoxanthine	1.8226	0.86597	0.000121
Pyridine-2-3-dicarboxylate	0.94216	-0.08596	0.000308
3-Sulfocatechol	0.44587	-1.1653	0.000315
L-Carnitine	0.75435	-0.40669	0.000327
D-Glucose	0.23628	-2.0814	0.000358
gamma-Glutamylcysteine	0.52818	-0.92091	0.000406
AMP	0.65715	-0.60571	0.00065
2,3-Diphosphoglyceric acid	2.1042	1.0733	0.000711
Stearidonic acid	0.49113	-1.0258	0.000929

(5-L-Glutamyl)-L-glutamine	0.66039	-0.5986	0.000956
(7S-8S)-DiHODE	0.73445	-0.44527	0.000958
Indole	0.91835	-0.12289	0.000984
2-Hydroxyglutarate	0.7868	-0.34592	0.001157
Allantoate	1.2291	0.29764	0.001355
Glycine	0.86836	-0.20363	0.001699
acyl-C14 (O-tetradecanoyl-L-carnitine)	0.74325	-0.42808	0.002012
L-Methionine	0.81413	-0.29666	0.002127
L-Adrenaline	0.72063	-0.47267	0.002386
L-Serine	0.87941	-0.18539	0.002397
acyl-C18 (Octadecanoyl-L-carnitine)	0.84291	-0.24654	0.003093
Spermine	0.80042	-0.32116	0.003377
Octadecanoic acid	0.83363	-0.26253	0.004629
3-Methyldioxyindole	0.89208	-0.16475	0.005146
Creatine	0.85633	-0.22376	0.005497
N-Methylethanolamine phosphate	0.85197	-0.23112	0.005636
1-4-beta-D-Xylan	1.3014	0.38001	0.007825
Decanoic acid	0.89069	-0.16701	0.00803
Glycochenodeoxycholate	1.5713	0.65198	0.00887
Sphingosine 1-phosphate	1.1798	0.23854	0.010068
2-3-Dinor-8-iso prostaglandin F2alpha	1.313	0.39286	0.011203
trans-4-Hydroxy-L-proline	0.82995	-0.2689	0.011888
L-Kynurenine	0.81177	-0.30086	0.011934
Inosine	0.63126	-0.66369	0.01256
UDP-N-acetyl-D-glucosamine	0.78651	-0.34645	0.022518
L-Proline	1.1799	0.23865	0.023867
Nonanoic acid	0.89729	-0.15636	0.024894
Adenine	0.93344	-0.09937	0.027107
Cortisol	0.72458	-0.46479	0.029102
Glycolate	0.91839	-0.12283	0.031042
D-Fructose 1-6-bisphosphate	0.75889	-0.39803	0.031491
2-Oxoglutarate	0.34873	-1.5198	0.034981
L-Valine	0.93735	-0.09334	0.036253
Dodecanoic acid	0.8707	-0.19975	0.044711
D-Ribose	0.89704	-0.15676	0.045216
Ectoine	1.534	0.61729	0.047602
acyl-C16 (L-Palmitoylcarnitine)	0.89449	-0.16087	0.049721

Supplementary Table 2 The [$^{13}C_6$] glucose and [1′,2′,3′,4′,5′-$^{13}C_5$] inosine tracer in erythrocytes of patients and healthy controls

Fraction	Duration	Glucose tracer		Inosine tracer		P value	P value	P value	P Value
		NC[1] (mean)	G[2] (mean)	NC[3] (mean)	G[4] (mean)	1 VS 3	2 VS 4	1 VS 2	3 VS 4
Glucose (M6)	0.5h	8.5%	7.8%	0.0%	0.0%	<0.001	<0.001	0.536	NA
	2h	5.3%	6.6%	0.0%	0.0%	<0.001	<0.001	0.213	NA
	6h	0.6%	2.7%	0.0%	0.0%	0.006	0.068	0.172	NA
F6P(M6)	0.5h	18.7%	10.7%	4.5%	19.0%	0.001	0.091	0.018	0.011
	2h	18.3%	13.6%	1.4%	18.8%	<0.001	0.403	0.151	0.009
	6h	19.5%	13.0%	2.8%	5.7%	<0.001	0.01	0.016	0.026
FBP(M6)	0.5h	22.0%	12.7%	9.8%	16.2%	0.008	0.325	0.011	0.112
	2h	32.2%	29.2%	5.1%	32.4%	<0.001	0.594	0.273	0.002
	6h	40.7%	38.2%	17.1%	16.0%	<0.001	<0.001	0.439	0.744
G3P(M3)	0.5h	28.2%	32.3%	25.2%	36.2%	0.318	0.553	0.305	0.05
	2h	45.9%	38.0%	22.6%	44.5%	0.002	0.399	0.016	0.033
	6h	61.2%	51.7%	48.9%	41.9%	<0.001	0.039	0.008	0.095
2,3-BPG(M3)	0.5h	12.5%	3.3%	14.2%	13.2%	0.388	0.001	0.004	0.589
	2h	39.8%	26.0%	17.8%	42.0%	0.001	0.029	0.001	0.007
	6h	62.6%	52.1%	58.6%	48.7%	0.17	0.598	0.063	0.082
3-PG(M3)	0.5h	13.8%	4.8%	15.4%	18.9%	0.383	0.001	0.004	0.172
	2h	42.0%	28.1%	17.5%	44.5%	0.005	0.02	<0.001	0.005
	6h	66.0%	53.8%	61.6%	49.7%	0.099	0.569	0.05	0.058
PEP(M3)	0.5h	11.9%	4.7%	13.5%	14.9%	0.381	0.003	0.006	0.567
	2h	38.8%	25.9%	16.6%	41.6%	0.005	0.029	0.001	0.007
	6h	63.7%	52.8%	59.3%	48.6%	0.144	0.544	0.059	0.095
Pyruvate(M3)	0.5h	4.1%	2.0%	5.7%	3.6%	0.055	0.016	0.007	0.023
	2h	12.8%	8.3%	7.1%	11.7%	0.004	0.042	0.004	0.021
	6h	19.5%	18.0%	15.7%	16.9%	0.057	0.758	0.629	0.699
Lactate(M3)	0.5h	7.2%	5.1%	5.5%	2.1%	0.055	0.003	0.025	0.001
	2h	17.5%	8.1%	11.8%	11.8%	0.114	0.027	<0.001	0.998
	6h	36.7%	22.6%	22.2%	24.0%	<0.001	0.695	0.006	0.115
6-PG(M6)	0.5h	9.1%	4.5%	2.7%	6.2%	0.003	0.414	0.022	0.098
	2h	9.8%	5.2%	0.1%	3.0%	<0.001	0.287	0.049	0.147
	6h	7.0%	4.8%	2.5%	1.6%	0.001	0.105	0.245	0.38
R5P(M5)	0.5h	15.0%	13.5%	28.8%	36.5%	0.006	<0.001	0.747	0.103
	2h	24.0%	26.3%	23.2%	38.5%	0.834	0.028	0.497	0.014
	6h	29.6%	32.3%	54.2%	47.0%	<0.0001	0.052	0.524	0.278
Sed7P(M7)	0.5h	10.3%	4.5%	16.7%	25.6%	0.094	<0.001	0.027	0.088
	2h	15.7%	13.3%	9.5%	22.1%	0.047	0.096	0.305	0.031
	6h	19.1%	17.1%	38.8%	34.6%	<0.0001	0.005	0.436	0.345
Ery4P(M4)	0.5h	4.9%	3.1%	3.8%	9.1%	0.562	0.053	0.384	0.075
	2h	2.3%	4.0%	1.6%	7.0%	0.329	0.286	0.144	0.064
	6h	5.1%	4.6%	5.9%	7.2%	0.348	0.191	0.634	0.479

Inosine(M5)	0.5h	27.9%	19.7%	80.4%	85.2%	<0.001	<0.001	0.456	0.004
	2h	21.9%	31.4%	76.7%	81.7%	<0.001	<0.001	0.214	0.018
	6h	27.7%	31.4%	71.8%	73.8%	<0.001	<0.001	0.471	0.407

The [$^{13}C_6$] glucose and [1′,2′,3′,4′,5′-$^{13}C_5$] inosine tracer were performed among four groups with the same concentrations. The first and the second groups were the glucose tracer in erythrocytes of healthy controls (NC) or glaucoma patients (G), while the third and the fourth groups were the inosine tracer in erythrocytes of healthy controls or glaucoma. P values were assessed among the four groups by using a two-tailed unpaired t-test. F6P, fructose-6-phosphate; FBP, fructose 1,6-bisphosphate; G3P, glyceraldehyde 3-phosphate; 2,3-BPG, 2,3-bisphosphoglycerate; 3-PG,3-phosphoglycerate; PEP, phosphoenolpyruvate; 6-PG, 6-phospho-D-gluconate;R5P, ribose-5-phosphate; Sed7P, Sedoheptulose 7-phosphate; Ery4P, erythrose 4-phosphate.

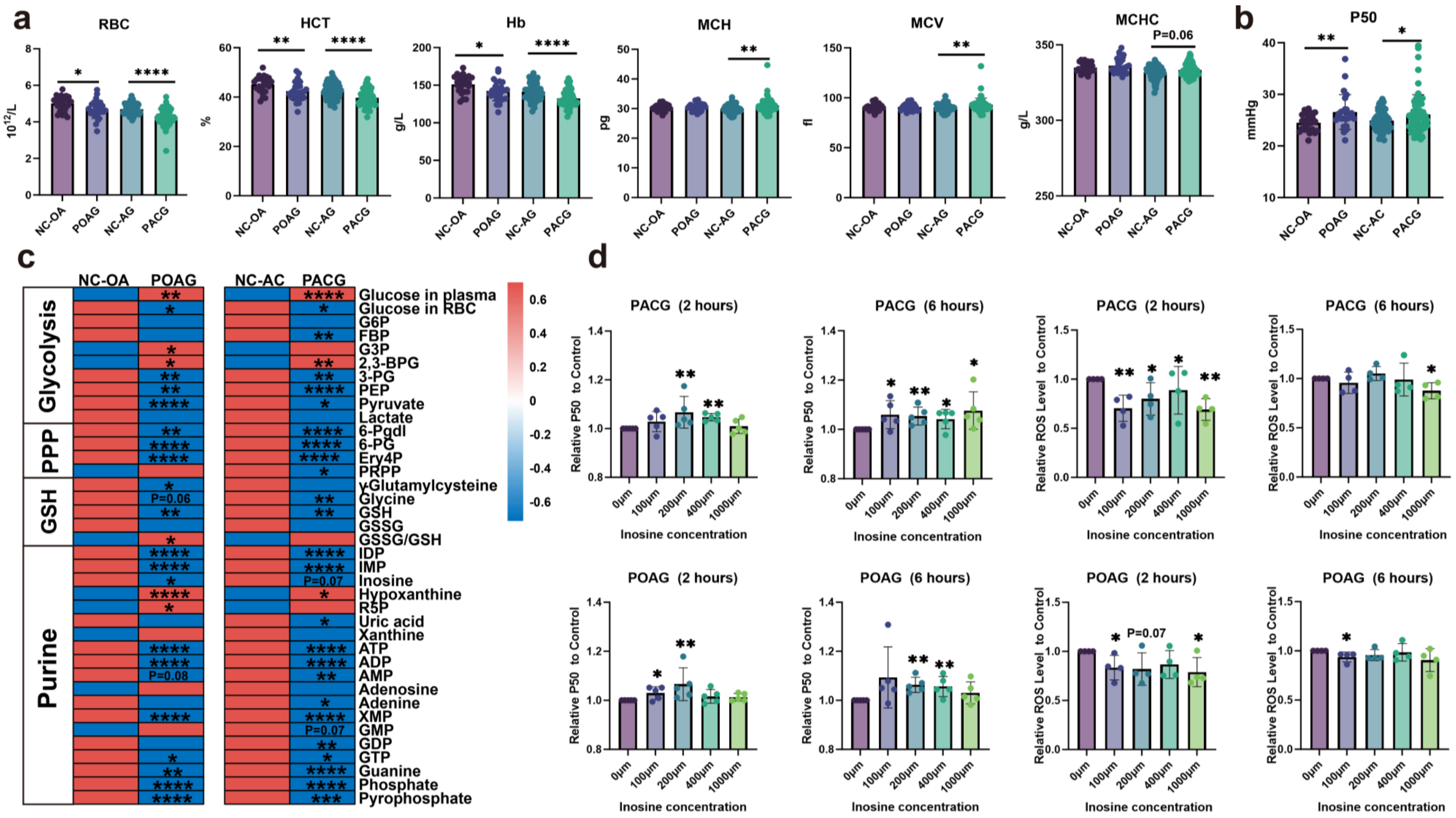
a
RBC
HCT
Hb
MCH
MCV
MCHC
P=0.06
b
P50
NC-OA
POAG
NC-AG
PACG
NC-AC
mmHg
c
NC-OA
POAG
NC-AC
PACG
Glycolysis
PPP
GSH
Purine
Glucose in plasma
Glucose in RBC
G6P
FBP
G3P
2,3-BPG
3-PG
PEP
Pyruvate
Lactate
6-Pgdl
6-PG
Ery4P
PRPP
γGlutamylcysteine
Glycine
GSH
GSSG
GSSG/GSH
IDP
IMP
Inosine
Hypoxanthine
R5P
Uric acid
Xanthine
ATP
ADP
AMP
Adenosine
Adenine
XMP
GMP
GDP
GTP
Guanine
Phosphate
Pyrophosphate
P=0.06
P=0.07
P=0.08
P=0.07
d
PACG (2 hours)
PACG (6 hours)
POAG (2 hours)
POAG (6 hours)
Relative P50 to Control
Relative ROS Level to Control
Inosine concentration
0μm
100μm
200μm
400μm
1000μm
P=0.07

Supplementary Figure 1. The changes in the number, function, and metabolism of erythrocytes among different subtypes of glaucoma

(a) Comparison of erythrocyte-related parameters between healthy controls and patients with primary open-angle glaucoma (POAG) and primary angle-closure glaucoma (PACG). **(b)** Comparison of the P50 value of erythrocytes between healthy controls and patients with POAG and PACG. **(c)** Heatmap of the erythrocyte intermediates of glycolysis, the pentose phosphate pathway (PPP), the glutathione synthesis pathway, and purine metabolism. Red represents an upregulated level, and blue represents a downregulated level. **(d)** The P50 and ROS levels of different glaucomatous erythrocytes treated with inosine at different concentrations. RBC, red blood cell; HCT, hematocrit; Hb, hemoglobin; MCV, mean corpuscular volume; MCH, mean corpuscular hemoglobin; MCHC, mean corpuscular hemoglobin concentration; NC-OA, the age- and sex- matched healthy controls for the patients with POAG; NA-AC, the age- and sex- matched healthy controls for the patients with PACG; G6P, glucose 6-phosphate; FBP, fructose 1,6-bisphosphate; G3P, glyceraldehyde 3-phosphate; 2,3-BPG, 2,3-bisphosphoglycerate; 3-PG, 3-phosphoglycerate; PEP, phosphoenolpyruvate; 6-Pgdl, glucono-1,5-lactone 6-phosphate; 6-PG, 6-phospho-D-gluconate; Ery4P, erythrose 4-phosphate; PRPP, phosphoribosyl pyrophosphate; GSH, glutathione; GSSG, glutathione disulfide; IDP, Inosine diphosphate; IMP, Inosinic acid; R5P, ribose 5-phosphate. Data are presented as mean ± SD; *$P<0.05$, **$P<0.01$, ***$P<0.001$.
